# Constructing a supercapacitor-memristor through non-linear ion transport in MOF nanochannels

**DOI:** 10.1093/nsr/nwae322

**Published:** 2024-09-11

**Authors:** Pei Tang, Pengwei Jing, Zhiyuan Luo, Kekang Liu, Xiaoxi Zhao, Yining Lao, Qianqian Yao, Chuyi Zhong, Qingfeng Fu, Jian Zhu, Yanghui Liu, Qingyun Dou, Xingbin Yan

**Affiliations:** Department of Materials Science and Engineering, Sun Yat-sen University, Guangzhou 510275, China; Department of Materials Science and Engineering, Sun Yat-sen University, Guangzhou 510275, China; School of Materials, Sun Yat-sen University, Shenzhen 518107, China; School of Materials, Sun Yat-sen University, Shenzhen 518107, China; Laboratory of Clean Energy Chemistry and Materials, Lanzhou Institute of Chemical Physics, Chinese Academy of Sciences, Lanzhou 730000, China; Department of Materials Science and Engineering, Sun Yat-sen University, Guangzhou 510275, China; Department of Materials Science and Engineering, Sun Yat-sen University, Guangzhou 510275, China; Department of Materials Science and Engineering, Sun Yat-sen University, Guangzhou 510275, China; College of Materials Science and Engineering, Hunan University, Changsha 410082, China; Department of Materials Science and Engineering, Sun Yat-sen University, Guangzhou 510275, China; School of Materials, Sun Yat-sen University, Shenzhen 518107, China; Department of Materials Science and Engineering, Sun Yat-sen University, Guangzhou 510275, China; Department of Materials Science and Engineering, Sun Yat-sen University, Guangzhou 510275, China

**Keywords:** supercapacitor, fluidic memristor, ionic hysteresis, resistive switching, non-linear curve

## Abstract

The coexistence and coupling of capacitive and memristive effects have been an important subject of scientific interest. While the capacitive effect in memristors has been extensively studied, the reciprocal scenario of the memristive effect in capacitors remains unexplored. In this study, we introduce a supercapacitor-memristor (CAPistor) concept, which is constructed by leveraging non-linear ion transport within the pores of a metal-organic framework zeolitic-imidazolate framework (ZIF-7). Within the nanochannels of the ZIF-7 electrode in an aqueous pseudocapacitor, the anionic species (OH^−^) of the electrolyte can be enriched and dissipated in different voltage regimes. This difference leads to a hysteresis effect in ion conductivity, constituting a memristive behavior in the pseudocapacitor. Thus, the pseudocapacitor-converted CAPistor seamlessly integrates the programmable resistance and memory functions of an ionic memristor into a supercapacitor, demonstrating enormous potential to extend the traditional energy storage applications of supercapacitors into emerging fields, including biomimetic nanofluidic ionics and neuromorphic computing.

## Teaser

This work introduces a supercapacitor-memristor concept that leverages non-linear ion transport within ZIF-7 frameworks, enabling rapid ion storage and memory functions, and opening new avenues in the field of iontronics.

## INTRODUCTION

**Figure 1.**
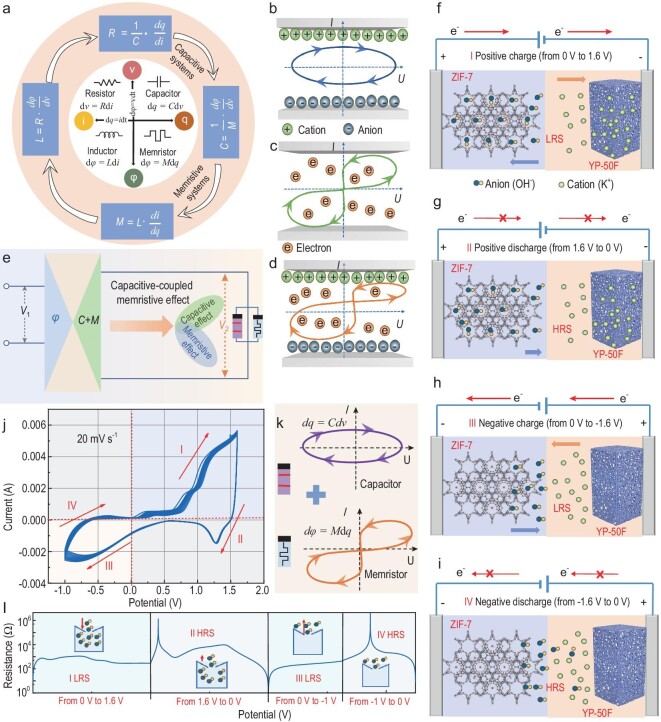
Capacitive-coupled memristive behavior and schematic illustration of the working mechanism of the CAPistor. (a) The relationship between fundamental circuit elements and their mathematical equations. (b) Current-voltage (I-U) curve in a capacitive system. (c) I-U curve in a memristive system. (d) The non-zero crossing I-U curve induced by capacitive effect in a memristive system. (e) A circuit diagram representing a parallel connection of a capacitor and a memristor. (f and g) The working electrode ZIF-7 is positively charged and the counter electrode negatively charged (forward bias, U > 0). (h and i) The working electrode ZIF-7 is negatively charged and the counter electrode positively charged (reverse bias, U < 0). (j and k) Schematic representation of the I-U curve of the CAPistor with coupling capacitance and memristor effects. During the test, the period of the voltage scan is 0 V→1.6 V→−1.0 V→0 V, and the scan rate is 0.02 V s^−1^. (l) Resistive switching behavior of the CAPistor in four voltage ranges.

The four electronic variables, charge, flux, current and voltage, can be combined two by two to form six relationships, corresponding to familiar passive devices such as resistors, capacitors and inductors. Among these relationships, only the relationship between charge and flux lacks clear articulation (Fig. 1a) [1]. In 1971, Chua advanced a theoretical perspective that introduced the concept of the fourth fundamental circuit element, the memristor, to describe the intricate interplay between charge and magnetic flux [2]. This theoretical proposition prompted further investigation and resulted in a significant breakthrough in 2008, namely the development of the world's first memristor prototype device by Hewlett-Packard Laboratory, utilizing titanium dioxide as the base material [1]. The advent of the memristor not only marked a paradigm shift in electronic components, but also opened a new avenue for advanced circuit design and computing paradigms.

The coexistence of any two fundamental circuit components, namely resistance (*R*), capacitance (*C*), inductance (*L*) and memristance (*M*), is a theoretical possibility. This coexistence can be elucidated through mathematical equations ($R = \frac{1}{C}\cdot\frac{{dq}}{{di}};\ L = R\cdot\frac{{d\varphi }}{{dv}};M = L\cdot\frac{{di}}{{dq}}$), illustrating their intricate interrelationships (Fig. 1a). It is evident that a single device can display both capacitive and memristive effects (*C* =$\ \frac{1}{M}$·$\frac{{d\varphi }}{{dv}}$). A memristor consists of two metal electrodes and a conductive active layer filled with anions and cations [3]. When subjected to an external electric field, it exhibits a capacitive effect (Fig. 1b–d) [4,5]. In the absence of an electric field, the persistent internal current, driven by the capacitive effect, results in a non-zero crossing in the current-voltage (I-V) curve. The phenomenon in question can be elucidated by a proposed capacitive-coupled memristive effect, depicted by an equivalent circuit model featuring an ideal memristor and a parallel capacitor (Fig. 1e) [4]. This model interprets the non-zero-crossing I-V hysteresis loop as a linear combination of capacitor and memristor currents, and the crossing point depends on the scanning direction [4,5]. It should be noted that although capacitive effects in memristors have been extensively reported, the exploration of the memristive effect in capacitors remains in its early stages.

Conventional electronics rely on electron flow, whereas iontronics, which draws inspiration from biological ion channels, employs nano-confined pore structures to regulate ion transport behavior in an intelligent manner [6]. Fluidic memristors take anions and cations in solution as carriers, and this ionic current carries a rich amount of chemical information, thereby providing key support for efficient parallel computing [7]. Inspired by the operating principles of biological ion channels, recent advances in fluidic memristors have used nanochannels for the precise control of ions in aqueous environments [8–11]. For example, Xiong *et al*. constructed an ionic memristor based on confined polyelectrolyte-ion interactions [8]. Robin *et al*. pioneered nanofluidic devices that feature nanometer-thick, 2D slits filled with salt solutions [12]. These innovative memristors operate at a voltage and power consumption analogous to those of biological systems, enabling precise control of ion flow through spatial confinement and molecular recognition.

Fluidic memristors and supercapacitors both rely on ion transport for their operation. Fluidic memristors use ion movement through a fluid, while supercapacitors store charge by adsorbing ions or through redox reactions on electrode surfaces [13–17]. Supercapacitors offer distinct advantages in terms of high power density, rapid response and extended lifetime, thereby providing crucial applications for electronic devices, aerospace, transportation and smart grids [18–20]. Recently, a kind of novel capacitive-type ionic device, the supercapacitor-diode (CAPode), has been reported [21–24]. Such devices limit free ion transport in supercapacitors to purposeful and selective unidirectional ion transport [25]. Fluidic memristors and supercapacitors both rely on ion transport mechanisms. In supercapacitors, charge is stored by adsorbing ions or through rapid redox reactions at the electrode surface, similar to how fluidic memristors operate [26]. Two types of iontronics concepts, fluidic memristors and supercapacitor diodes, were proposed, which led us to a bold idea: could a supercapacitor be endowed with memory performance comparable to that of a fluidic memristor through the design of nano-ion channels inside the electrode material? If yes, besides the traditional energy storage function, the supercapacitor would realize hysteresis of the transport and redistribution of electrolyte ions under a changing electric field.

In this work, we propose the concept of a supercapacitor-memristor (CAPistor), by converting a simple asymmetric pseudocapacitor structure (a metal-organic-framework (MOF) material-zeolitic imidazolate framework ZIF-7 is used as the working electrode, a commercially available activated carbon−YP-50F is used as the counter electrode, and potassium hydroxide (KOH) solution is used as the aqueous electrolyte) into an ionic memristor with a high ON/OFF ratio (5349 at 20 mV s^−1^). The success of this conversion is contingent upon the enrichment and dissipation of the anions (OH^−^) in the pseudocapacitor at varying voltages within the nanochannels of the ZIF-7 electrode across disparate voltage ranges of the non-linear curve. This phenomenon gives rise to a hysteresis effect in ionic conductivity and the formation of a memristive characteristic. The resistance switching behavior is demonstrated using density functional theory (DFT) calculations and X-ray absorption fine structure (XAFS). Furthermore, the CAPistor exhibits advantages such as high speed, low power consumption and long lifespan, making it promising for emerging fields, including neuromorphic computing and artificial synapses.

## RESULTS AND DISCUSSION

### Design principles of the CAPistor

A novel CAPistor was constructed using ZIF-7 and commercially available activated carbon YP-50F as the two electrodes and 1 M KOH as the electrolyte (detailed material characterizations are provided in Note S1 and Figs S1–S8). According to the working mechanism, four cases should be considered. (i) During the application of a positive charge to the ZIF-7 electrode and gradual increase of forward bias, OH^−^ ions enter the pores of ZIF-7 while K^+^ ions are adsorbed onto the surface of YP-50F (Fig. 1f). This results in a significant current response and circuit conduction. (ii) As the forward bias gradually decreases, a very small amount of OH^−^ is released from the pores of the zeolite imidazolium framework (Fig. 1g). This establishes an ion concentration equilibrium in the pores, while the external electric field balance lags behind, resulting in a minimal current response. (iii) When the ZIF-7 electrode is negatively charged and the reverse bias gradually increases, the remaining OH^−^ overcomes the potential barrier (Fig. 1h), resulting in a significant current response. (iv) As the reverse bias gradually decreases, there is no ion movement in the ZIF-7 electrode pores (Fig. 1i), resulting in an open circuit and no current response.

The physics of fluidic memristors reported so far stems from the behavior of electrolytes under nanoscale confinement. The switching between their high/low conductance states involves two different mechanisms. One mechanism is the formation of Bjerrum ion pairs under confinement conditions, as proposed by Bocquet *et al.* [12]. At low voltages, these ion pairs are restrained, leading to a reduction in ionic conductance, while at higher voltages, these ion pairs are destroyed and ionic conductance is restored. Another mechanism was discovered by Xiong *et al.* [8]. They noted that the large surface charge density of the polyelectrolyte brushes slowed down the equilibrium velocity of the ions, resulting in a non-linear hysteretic loop. Thus, the origin of the hysteresis return line in the CAPistor may be similar to that of fluidic memristors. We will discuss the potential mechanisms behind these observations in detail.

Figure1j shows that the CAPistor exhibits a non-linear I-V characteristic when probed by a time-varying bias voltage. This characteristic does not exceed the zero point and is similar to that of the recently reported fluidic memristor [10]. Additionally, the curve satisfies the history-dependent memristor property, where the hysteresis curve shows a non-zero current when the potential is zero. On the one hand, according to Chua's theory, the compressed I-V curves at periodically applied voltages exhibit a non-zero-crossing feature, consistent with history-dependent memristor properties [8]. This offset arises from the effect of surface charges in asymmetric channels, a phenomenon usually observed in biological memristors (e.g. K^+^ ion channels) [8]. On the other hand, the CAPistor inherently adopts a sandwich structure in which the internal electric field still exists when the external voltage drops to zero, which is due to capacitive effects. Therefore, the current-voltage (I-U) hysteresis behavior of this non-linear memristor is equivalent to the sum of the capacitance-current and memristor-current in the indicated scan direction (Fig. 1k).

The I-V characteristics of the CAPistor demonstrate significant resistive switching effects. As shown in Fig. 1l, the device's resistive state changes from its original state to a low-resistance state (LRS) when the applied potential gradually increases to 1.6 V. When the applied potential is decreased from 1.6 V to 0 V, the response current becomes extremely small and switches from the low-resistance state to the high-resistance state (HRS). In the hysteresis curve, a non-zero current is observed when the voltage is zero. When the applied voltage sweeps to about −1.0 V, the device repeatedly resets from LRS to HRS.

The CAPistor demonstrates non-linear ion transport within the nanochannels of ZIF-7 during various potential stages. Ions exhibit different aggregation states on a non-linear curve, corresponding to a high-conductance enriched state (‘on’ state, denoted as ‘1’) and a low-conductance dissipative state (‘off’ state, denoted as ‘0’). Additionally, it is important to note that the transition between high and low conductance states is not immediate. Rather, it requires a certain duration, indicating a hysteresis effect in the ion state transition. As a result, the delayed transition in ion states leads to a memory effect in ion transport within the ZIF-7 nanochannels, which allows for the retention of previous ion states.

### Electrochemical and ‘memristive’ performance of the CAPistor

**Figure 2.**
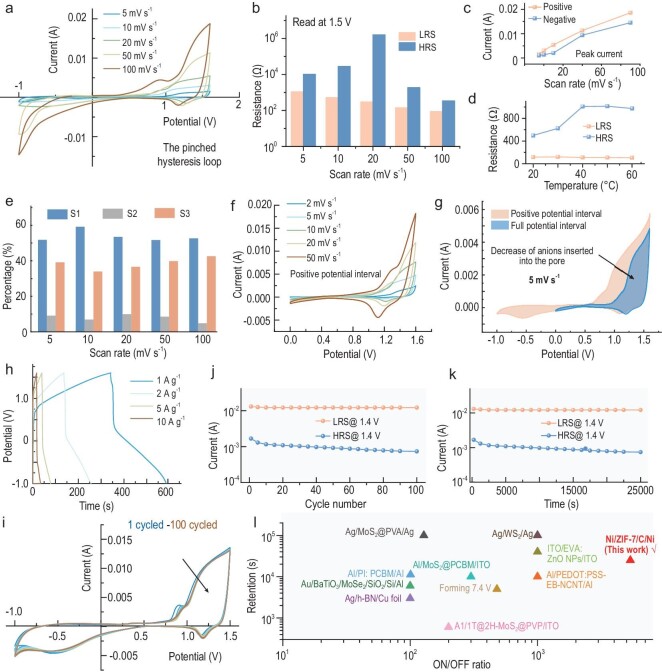
Electrochemical and memristive performance of the CAPistor. (a) CVs at different scan rates. (b) The scan rate dependence of resistances read at 1.5 V in HRS and LRS. (c) Peak current at different scan rates. (d) The temperature dependence of resistances read at 1.5 V in HRS and LRS. (e) Percentage of area in different quadrants of the CV curve. (f) CV curves for positive voltage intervals. (g) CV curves at 5 mV s^−1^ for positive voltage intervals vs. full voltage intervals. (h) GCD curves at different current densities. (i) CV curves from the 1st to 100th cycle. (j) Current values read at 1.4 V in HRS and LRS for different numbers of cycles. (k) Current values read at 1.4 V in HRS and LRS for different times during the cycle. (l) Comparison of resistance switching performance (retention and ON/OFF ratio) of the CAPistor with other conventional memristors [28–37].

The dependence of the response current on the scan rate was first investigated. As demonstrated in Fig. 2a and Fig. S10, the I-U curves display non-linear characteristics for scan rates ranging from 5 to 100 mV s^−1^. Additionally, the peak current gradually increases with the scan rate (Fig. 2c). The presence of significant current response in the first and third parts produces the ‘pinched’ hysteresis loop under periodic forcing, which is characteristic of a memristor. Additionally, a weak reduction peak occurs at ∼1.3 V due to the removal of a small amount of OH^−^ anions from the MOF. As displayed in Fig. 2b, the resistance value of HRS is consistently higher than that of LRS (read at 1.5 V) at different scanning rates. In particular, the ON/OFF ratios were calculated at different scan rates (Fig. S11). The highest ON/OFF ratio is 5349 at 20 mV s^−1^, which is comparable to the resistive switching of conventional memristors [27]. Figure 2d shows that as the temperature increases from 20°C to 60°C, the resistance of the LRS decreases from 117 Ω to 106 Ω, while the resistance of the HRS increases from 500 Ω to 974 Ω. This confirms the feasibility of the device's operation in high-temperature environments, as the ON/OFF ratios of the CAPistor increase with increasing temperature (see Fig. S12 and Note S2 for detailed discussion).

The cyclic voltammetry (CV) curve is divided into four parts, as shown in Fig. 2a, and defined as S_1_ (positive charge from 0 V to 1.6 V), S_2_ (positive discharge from 1.6 V to 0 V) and S_3_ (negative charge from 0 V to −1.0 V). Typically, conventional pseudocapacitors only operate within the positive voltage interval, and the charge accumulated in the first part is usually fully discharged in the second part. In contrast, Fig. 2e shows that only a small portion of the charge accumulated by the CAPistor in the first part is released in the second part, while most of the charge is released in the third part. Specifically, S_1_ accounts for 50% to 60% of the total area of the CV curve at different scan rates, whereas S_2_ accounts for 5% to 10% and S_3_ accounts for 30% to 40% (see Note S3 for a detailed discussion). Therefore, the fluctuation in the applied voltage causes a dynamic modulation of the accumulation and dissipation states of OH^−^ anions within the nanochannels, resulting in non-linear ion transport.

The device's capacitive performance was evaluated in the positive voltage interval (Fig. 2f). Notably, the device displays a low coulombic efficiency, as shown by the strong oxidation peak compared to the reduction peak. Furthermore, the current response of the positively biased part is significantly increased when a negative voltage scan is applied (Fig. 2g). This phenomenon can be attributed to the non-released OH^−^ ions during the positive discharge process being released in large quantities after being subjected to negative bias. This provides excess active sites for the next cycling process during positive charging. Scanning negative is equivalent to regenerating the device after scanning positive (see Note S3 for detailed discussion). Thus, alternating negative and positive voltage scans of a conventional pseudocapacitor results in a compression hysteresis I-U curve that is similar to that observed in fluidic memristors. The galvanostatic charge-discharge (GCD) curves show that the charging capacity is much larger than the discharging capacity in the positive voltage interval (Fig. 2h), and most of the discharging capacity is in the negative voltage interval, confirming the results of the CV curves.

Cycle stability is crucial for the reliability and lifespan of fluidic memristors. While solid-state memristors have seen significant improvements in cycle stability over the past decade, fluidic memristors, still in early research stages, often lack sufficient cycle stability. The durability of the CAPistor was tested, and the device demonstrated excellent stability in a cycling test at 20 mV s^−1^ (Fig. 2i). There is little fluctuation in the current values in the HRS and the LRS after 100 scan cycles (Fig. 2j), indicating that the high switching ratio can be maintained for at least 25 000 s (Fig. 2k). This suggests that the resistive switching based on the CAPistor is non-volatile. When comparing the resistive switching performance of the CAPistor to that of organic and oxide-based memristors reported in previous studies [28–37], it is evident that the CAPistor has a higher ON/OFF ratio than most conventional memristors. Additionally, its endurance performance is comparable (reported solid-state memristors based on different mechanisms also have a cycle count of around 100 cycles), as shown in Fig. 2l and Table S1. In short, the CAPistor not only has advantages in ON/OFF ratio, but its cycling stability also reaches the level of existing solid-state memristors, demonstrating its potential in practical applications.

### Memristive mechanism in pseudocapacitors

**Figure 3.**
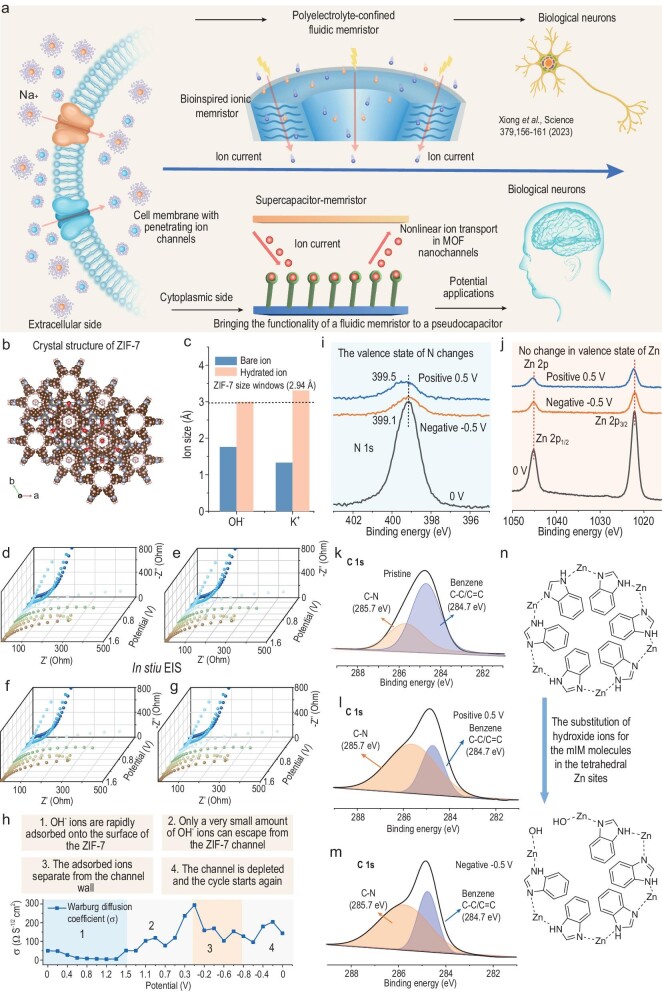
Unveiling the memristive mechanism in the pseudocapacitor. (a) Schematic illustration of ion transport memristive properties: from biological nanochannels to ionic memristor to CAPistor. (b) Crystal structure of ZIF-7. (c) Comparison of anionic and cationic sizes in KOH electrolyte with the ZIF-7 size window. (d–g) Nyquist plots of the CAPistor at different potentials. (h) Response of the Warburg diffusion coefficients (σ) to the applied potential. (i and j) N 1s nd Zn 2p XPS spectra of the ZIF-7 electrode at different voltages. (k–m) C 1s XPS spectra of the ZIF-7 electrode at different voltages. (n) Schematic diagram of the structural evolution of the ZIF-7 electrode under different voltages.

Nanochannels regulate ion transport in cells, which is critical to life processes. The memristive phenomenon is a memory effect in which the resistance of biological ion channels changes in response to external signals (Fig. 3a). Emulating nature can lead to energy-efficient and efficient computing architectures. In recent years, fluidic memristors based on ion-confined pore channels have emerged [10,38]. The surface charge of nanochannels and their induced double electric layer, under the action of an applied varying electric field, causes hysteresis of ion transport or redistribution in nanochannels. This leads to a memory effect in nanochannel resistance [7,9].

The CAPistor combines the energy storage capabilities of supercapacitors with memristive properties, offering superior energy density and efficiency compared to fluidic memristors. Design of a supercapacitor sandwich structure ensures enhanced durability and stability, avoiding issues like evaporation and leakage, thereby extending device lifespan and ensuring reliable operation in diverse environmental conditions. Developing a bio-inspired supercapacitor-memristor could enable the design of artificial nanofluidic chips for neuromorphic computing. This technological advancement could enable more efficient computing architectures and neural network simulations.

ZIF-7 is a well-known MOF material [39]. It belongs to the class of zeolite multihollow skeletal materials, where bridging oxygens in zeolite molecular sieves are replaced by imidazoles and their derivatives connected to the N atoms in the imidazoles by metal ions [40,41] (Fig. 3b). As shown in Fig. 3c, the pore window size of ZIF-7 is 2.9 Å, which is larger than the size of OH^−^ anions and K^+^ cations. This suggests that the ions can easily enter the pore. To differentiate the roles of cations and anions in the resistive switching mechanism, we constructed asymmetric ionic components using the other two electrolytes (0.5 M K_2_SO_4_ and 1.0 M NaOH). In the NaOH electrolyte (Fig. S23), the CV curves exhibit a ‘pinched’ hysteresis loop, which is analogous to those observed in the KOH electrolyte. However, the hysteresis loop disappears in the K_2_SO_4_ electrolyte (Fig. S24a). Moreover, the CV curve area in K_2_SO_4_ is significantly smaller than in the other two electrolytes (Fig. S24b), suggesting that K^+^ ions play a minimal role in electrochemical capacity. Based on the available evidence, it can be concluded that the memristive effect in the CAPistor is a result of OH^−^ anion transport hysteresis in ZIF-7 pore channels, which appears to be independent of K^+^ ions.

Electrochemical impedance spectra (EIS) were recorded under positive and negative polarization to analyze ion transport in the CAPistor in dynamic conditions. The EIS curves show a 45° Warburg region in the mid-frequency region and a significant deviation from the vertical line in the low-frequency region (Fig. 3d–g). This deviation can be attributed to the pseudocapacitance associated with the finite-length diffusion of the OH^−^ anion in the ZIF-7 electrode material [42,43]. The Warburg diffusion coefficient (σ) is further measured by the slope of the real Z versus *ω*^−1/2^ (see Note S5 for detailed discussion) [44]. As shown in Fig. 3h, when the potential of the CAPistor is gradually increased from 0 V to 1.5 V, the σ value gradually decreases from 49.5 Ω S^−1/2^ cm^2^ to 5.5 Ω S^−1/2^ cm^2^. This indicates that the diffusion coefficient of OH^−^ in the channel gradually increases, driven by the applied potential, which corresponds well with the results of the CV curves. In contrast, when discharging from 1.5 V to 0 V, the σ value increased significantly (from 50.6 Ω S^−1/2^ cm^2^ to 294.3 Ω S^−1/2^ cm^2^), implying that the rate of OH^−^ ion removal in the channel is relatively slow. During the process of charging negatively from 0 V to −1 V, the σ value decreased from 294.3 Ω S^−1/2^ cm^2^ to 95.4 Ω S^−1/2^ cm^2^. This indicates that OH^−^ is able to successfully desorb from the channel in the potential interval, which coincides with the significant response currents appearing in the CV curves. When discharging negatively from −1.0 V to 0 V, the σ values remain roughly in the range of 100 Ω S^−1/2^ cm^2^ to 200 Ω S^−1/2^ cm^2^, and there are no ion insertion and extraction processes.

The mechanism of charge storage for the ZIF-7 electrode and the memristive effect in the pseudocapacitor were further investigated by *ex-situ* X-ray photoelectron spectroscopy (XPS). The N 1s spectrum in Fig. 3i shows that the position of the N peak increased from 399.1 eV to 399.5 eV after the ZIF-7 electrode was biased in the forward direction, indicating an increase in the valence state of N during the charging process [45]. In contrast, when a negative voltage is applied, the peak position of N remains at 399.1 eV, indicating that the valence state of N remains unchanged. Additionally, the position of the Zn 2p peak remains constant whether a positive or negative bias is applied (Fig. 3j), demonstrating that the valence state of Zn remains unchanged. Therefore, the redox reaction of ZIF-7 in alkaline electrolyte relies on the valence change of N rather than the transition metal element Zn. The C 1s spectrum in the ZIF-7 electrode displays peak positions at 284.7 eV (P_1_) and 285.7 eV (P_2_), corresponding to C−C/C=C and C−N in the benzene ring, respectively [45] (Fig. 3k). Notably, the area of the peak located at 284.7 eV decreases significantly when the electrode is subjected to a positive voltage (Fig. 3l). The absence of part of the benzene ring can be attributed to the substitution of hydroxide ions for the mIM molecules on the tetrahedral Zn sites [46]. However, the change in the region of the C 1s spectrum when subjected to a negative voltage is not very significant compared to that of the positive voltage (Fig. 3m), suggesting that the breakage of the N−Zn bond is not fully reversible. Furthermore, when exposed to a positive bias voltage of 0.5 V, the C 1s peak increased from 284.7 eV to 284.9 eV (Fig. S27). This increase is attributed to the substitution of hydroxide ions for mIM molecules on the tetrahedral Zn sites (Fig. 3n), which results in the breakage of the N−Zn bonds between part of the benzene rings. The breaking of the N−Zn bond resulted in the strengthening of the N−C bond outside the benzene ring and the C−C bond inside the benzene ring. As a result, the C 1s peak increased by 0.2 eV and the N 1s peak increased by 0.4 eV.

**Figure 4.**
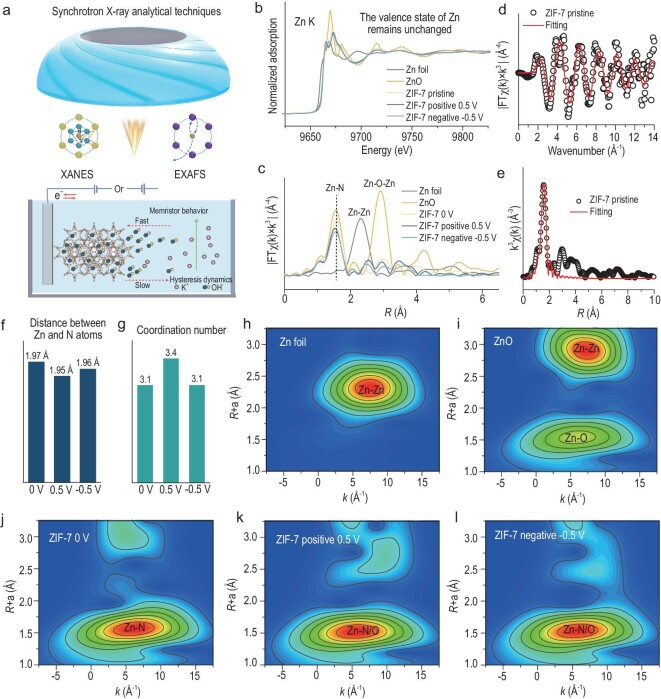
XAFS characterizes the atomic and electronic structure of the ZIF-7 electrode. (a) Schematic diagram of the structural evolution of ZIF-7 in an electrochemical process investigated by synchrotron radiation characterization. (b) XANES spectra of Zn foil, ZnO and the ZIF-7 electrode at different voltages. (c) EXAFS of the ZIF-7 electrode at different voltages, Zn foil and ZnO. (d) EXAFS k^3^χ(k) spectra at the Zn K edge of the ZIF-7 electrode without applied voltage. (e) EXAFS k^3^χ(R) spectra and fitting results at the Zn K edge of the ZIF-7 electrode without applied voltage. (f) Variation of distance between Zn and N atoms from EXAFS spectra fitting. (g) Variation of coordination number of Zn atoms from EXAFS spectra fitting. (h–l) Zn K-edge wavelet transform extended X-ray absorption fine structure (WT-EXAFS) spectra of Zn foil, ZnO and the ZIF-7 electrode without applied voltage; ZIF-7 electrodes applying a positive voltage of 0.5 V; ZIF-7 electrodes applying a negative voltage of −0.5 V.

X-ray absorption near-edge structure (XANES) and extended X-ray absorption fine structure (EXAFS) were used to further determine the coordination environments and valence states of the ZIF-7 electrodes during positive and negative polarization (Fig. 4a). The K-edge XANES spectral edge of Zn in ZIF-7 almost coincides with ZnO, indicating that the valence state of Zn is +2 at different states (Fig. 4b), which confirms that Zn is not an electrochemically active center (in agreement with the XPS results discussed above). The EXAFS spectra indicate that the Zn is present in the ZIF-7 electrode mainly in the form of Zn−N bonds (Fig. 4c), and the fitting results are shown in Fig. 4d and e, and Figs S28–S32. When ZIF-7 is subjected to forward bias, the peak representing Zn−N/O coordination is shifted towards the left. At this time, the bond length of Zn−N/O decreases from 1.97 Å to 1.95 Å (Fig. 4f). In addition, the coordination number of Zn increases from 3.1 to 3.4 (Fig. 4g). It can be attributed to the adsorption of OH^−^ in the electrolyte to the Zn center atom, which increases part of the Zn−O bond. Thus, the bond of the Zn center atom is strengthened, leading to a shortening of the bond length. In contrast, when a negative bias voltage of −0.5 V is applied, OH^−^ desorbs from the Zn central atom and Zn−O coordination is absent, resulting in the coordination number recovering again from 3.4 to 3.1 and the bond length rising to 1.96 Å. The results of the wavelet transform are shown in Fig. 4h–l. As the wavelet transform patterns of Zn−N coordination and Zn−O coordination are extremely similar, the Zn center atom of ZIF-7 still maintains the Zn−N coordination structure during charging and discharging, although part of the Zn−O coordination has been introduced into the Zn center atom.

A fluidic memristor, as a passive non-linear device, is constructed based on the realization of non-linear ion transport under spatially constrained conditions. Researchers have conducted in-depth studies on anomalous ion transport phenomena under multi-scale spatial confinement, and have put the non-linear ion transport mechanisms into the following five categories [6]: (i) ionic coulomb blockade as a Wien effect; (ii) adsorption/desorption effects of ions in nanochannels; (iii) concentration polarization; (iv) hydrophobic gating; (v) structural deformation of the nanopores/channels. Of the five non-linear ion transport mechanisms mentioned above, the supercapacitor-memristor proposed in this study is mainly based on the ‘adsorption/desorption effects of ions in nanochannels’ (Fig. S33a). Specifically, the adsorption of anionic OH^−^ within the MOF framework alters the concentration distribution and thus affects ionic conductivity. The interaction between adsorption and desorption leads to a hysteresis phenomenon in the current-voltage characteristics of the supercapacitor-memristor. The adsorption process of OH^−^ inside the MOF pores is faster with lower energy barriers, while the desorption process is slower with higher energy barriers (Fig. S33b). Based on the *in-situ* EIS and *ex-situ* XAFS measurements, we can provisionally conclude that the memory effect in supercapacitors is caused by the non-linear ion transport of OH^−^ anions inside the ZIF-7 pore. The hysteresis of ion transport or redistribution causes the nanochannel to exhibit the memory effect.

### Density functional theory calculation method

**Figure 5.**
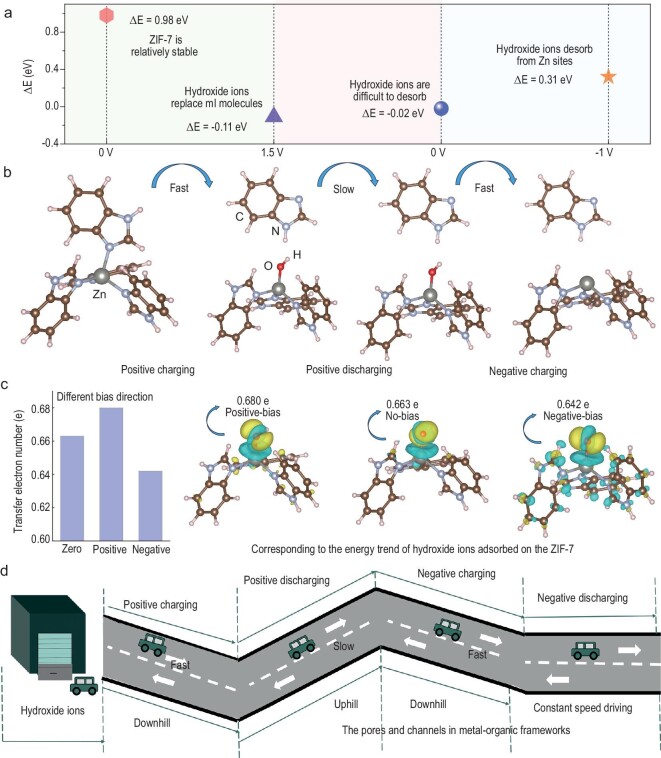
DFT calculations. (a) The adsorption energy of OH^−^ ions to the Zn site in the ZIF-7 electrode varies at different potentials. (b) Schematic representation of the evolution within the ZIF-7 crystal structure at different potentials. (c) Differential charge density difference. Iso-surface is 0.003e/A^3^. Yellow and blue regions represent electron accumulation and electron deletion lacking, respectively. (d) Schematic diagram for ion transport of OH^−^ in the channels of ZIF-7 at different potentials. The cars represent OH^−^ ions and the roadways represent channels in the ZIF-7 electrode.

To gain more insight into the memristive mechanism of the pseudocapacitor, we performed first-principles calculations using DFT to validate non-linear ion transport in MOF nanochannels. This was done to understand the ‘pinched’ hysteresis loop that appears in the I-U curves. As demonstrated in Fig. 5a, the direct dissociation of ZIF-7 without an applied electric field to break the bond results in an energy change (*ΔE*) of 0.98 eV, indicating that ZIF-7 is relatively stable. However, if a positive potential is applied to ZIF-7, hydroxide ions are driven by the external electric field into the pore interior and bond with the metal atom Zn at the center of the ligand (Fig. 5b). This allows the imidazole ligand to detach from the ZIF-7. This process results in an energy change of −0.11 eV, while the latter process results in a more negative energy change, indicating a faster rate of ion insertion into the pore. This faster rate corresponds with the significant response current of CAPistors’ I-U curve in the forward charging interval. Upon discharge to 0 V, there is an energy change of −0.02 eV. At this point, the rate of OH^−^ ion detachment from the pore is very slow due to the strong interaction between the hydroxide ions and the Zn atoms. This results in a very low current response during forward discharge. When under negative voltage, the corresponding *ΔE* is 0.31 eV, indicating that the hydroxyl group is difficult to adsorb in ZIF-7. In summary, a strong negative electric field destroys the stabilized bonding of OH^−^ with Zn atoms. Additionally, during the positive charging process, all hydroxyl groups accumulated in the pores will be desorbed, resulting in a significant response current during the negative charging interval. Only a small portion of hydroxyl groups is desorbed during the positive discharging process.

We then analyzed the differential charge density of ZIF-7 (iso-surface s 0.003e/A^3^), with the yellow and blue regions representing electron accumulation and electron lacking, respectively (Fig. 5c). The number of electrons transferred to the hydroxyl ligand in the positive-bias, zero-bias and negative-bias cases is 0.680 e, 0.663 e and 0.642 e, respectively, which corresponds to the energy trends of the three cases of OH^−^ ions adsorbed on the ZIF-7. It is clearly seen that the number of electrons transferred increases with positive bias and decreases with negative bias relative to zero bias. This statement is in line with the preceding assertion that as the positive voltage increases, hydroxide ions bond with the metal atom Zn at the center of the ligand. Conversely, as the negative voltage increases, hydroxide ions dissociate from the Zn metal atoms.

Based on the above discussion, we compare the OH^−^ transport in the ZIF-7 pore at different potentials to a car traveling on a road. The comparison is divided into four parts (Fig. 5d): (i) moving downhill at a high speed; (ii) moving uphill at a slow speed; (iii) moving downhill at a high speed; (iv) driving at a constant speed. The energy barrier for the insertion and extraction of Zn metal atoms and OH^−^ anions is a function of their interaction strength within the confined space, resulting in non-linear ion transport.

### The ‘forgetting-relearning-forgetting’ process

Supercapacitors operate by allowing ions to move rapidly between the anode and cathode, enabling the storage and release of energy [18]. The conductivity between the anode and cathode is determined by the number of ions in the channel, which represents the information stored in the device. The concept of the CAPistor presents the possibility of supercapacitors simulating memristor-based synaptic behavior.

**Figure 6.**
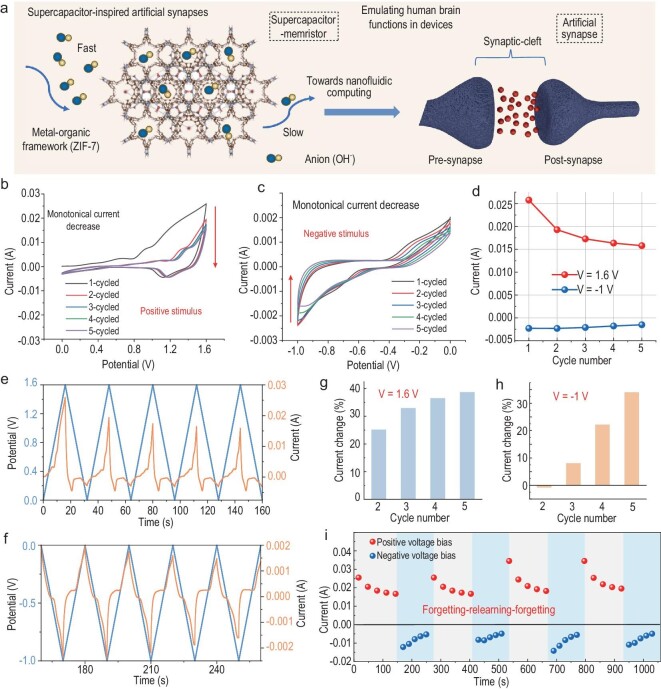
Electrical testing of the CAPistor under a voltage-sweeping algorithm. (a) Schematic diagram of the CAPistor towards synaptic function simulation. (b and c) The independent I-V characteristics of the CAPistor at positive voltage bias (0→1.6→0 V) and negative voltage bias (0→−1.0→0 V), respectively. (d) Current variations at 1.6 V or −1.0 V obtained from (b) and (c) are plotted as a function of cycle number. (e and f) Evolution of the current (orange) under positive and negative voltage pulses of constant polarity (blue). (g and h) Current change under continuous positive voltage stimulus (V = 1.6 V) and negative voltage stimulus (V = −1 V). (i) Time-evolving illustration of the ‘forgetting-relearning-forgetting’ process.

In biological systems, when a nerve impulse reaches a synapse, it opens channels that allow calcium ions to flow in. This influx of calcium triggers the release of neurotransmitters, which cross the synaptic cleft and transmit the impulse to the next neuron [7]. An artificial synapse is a novel electronic device that simulates interconnections between neurons to build neural networks and perform brain-like computational functions [8,10,12]. Mao *et al.* designed and fabricated a fluid channel confined by a polyimidazole brush (PimB) to control ion flux through spatial confinement and molecular recognition [8]. The device functioned as a natural memristor and successfully simulated a variety of neuroelectric impulse behaviors. As shown in Fig. 6a, the spatial confinement of ions within the ZIF-7 pore is utilized to slow down ionic equilibrium, and imparts hysteretic conductivity behavior and artificial synaptic functionality to the CAPistor.

Inspired by the memristive effect in supercapacitors, we preliminarily tested the progressive resistive behavior of a CAPistor in scanning voltage mode to emulate the synaptic behavior in biological neurons. The artificial synapses, which are based on supercapacitors, exhibit a gradient conductivity characteristic, as shown in Fig. 6b and c, when a unidirectional voltage scan is applied (from 0 V to 1.6 V and from 0 V to −1.0 V), indicating cumulative resistance change. The CAPistor has a rectification function, as evidenced by the fact that the corresponding currents for positive scans are much higher than those for negative scans (Fig. 6d). This feature provides the basis for the device to achieve unidirectional information delivery for artificial synapses [47]. Additionally, under continuous voltage stimulation, the CAPistor demonstrated a gradual and time-dependent trend of current reduction, as shown in Fig. 6e and f [8,12]. By adjusting the cumulative factor of voltage stimulation, variations in CAPistor response current can simulate neuronal synaptic weight changes. Figure 6g and h illustrate the current of the first run cycle (read at 1.6 V and −1 V), which can be seen as a characteristic of forgetting behavior. To reveal the forgetting behavioral characteristics in detail, we conducted a repeated running program. Figure 6i shows that the forgetting process always occurs under a unidirectional voltage bias, which is consistent with human memory characteristics [47]. The human brain typically experiences a rapid initial loss of memory, followed by a slower decay. Remarkably, the CAPistor follows the trend of memory loss in the human brain, which may help recall past forgetting processes over time [47]. After undergoing a period of negative voltage stimulation (five times, ∼100 seconds each), the CAPistor current can be restored to a higher level, indicating a process of relearning. In summary, the negative voltage region can provide a superior cognitive environment for the CAPistor, resulting in enhanced memory capacity. Conversely, positive voltage scans only allow for the random recall of some forgotten information, implying impaired memory retention.

Although the currently proposed CAPistor demonstrates potential as a key nanofluid iontronic device, significant challenges remain with regard to realizing its success in a wider range of applications. These challenges include the in-depth study and precise characterization of the ion transport mechanisms in the confined domains of nanofluids, as well as how to transition from current sandwich-structured devices to miniaturization at smaller scales. In addition, building complex and connectable nanofluidic circuits is a key challenge, requiring solving interconnection issues between multiple devices as well as stability and consistency challenges at small scales.

## CONCLUSION

In summary, the integration of the resistive switching characteristics of a fluidic memristor into a supercapacitor has resulted in a breakthrough CAPistor with fast ion storage and memory functions. Using DFT calculations together with XAFS characterization, we have systematically elucidated the crucial role of the non-linear ion transport and hysteresis dynamics of anions in ZIF-7 pores, providing crucial insights into the determinants of the resistive switching characteristics of the CAPistor. The CAPistor exhibits progressive conductance tuning under a scanning voltage mode, mimicking the synaptic behavior observed in biological neurons. Furthermore, repetitive stimulation of the CAPistor can establish a ‘forgeting-relearning-forgetting’ process. This study not only reveals the memristive effect in supercapacitors for the first time, but also significantly advances our fundamental understanding of the relationship between capacitive and memristive devices. The establishment of this research paradigm represents a significant advancement in the field of neuromorphic devices, offering a promising avenue for the development of next-generation iontronic systems.

## METHODS

Experimental details and characterizations are listed in Supplementary Data.

## Supplementary Material

nwae322_Supplemental_File
