# Supplementary material for: Constructing a supercapacitor-memristor through non-linear ion transport in MOF nanochannels
Source: Natl Sci Rev. 2024 Sep 11;11(10):nwae322. doi: 10.1093/nsr/nwae322 (PMC11462086; doi:10.1093/nsr/nwae322)
Supplement: nwae322_Supplemental_File [file nwae322_supplemental_file.pdf]

Constructing a Supercapacitor-Memristor through Nonlinear Ion Transport in MOF Nanochannels

Pei Tang[a], Pengwei Jing[a], Zhiyuan Luo[b], Kekang Liu[b], Xiaoxi Zhao[c], Yining Lao[a], Qianqian Yao[a], Chuyi Zhong[a], Qingfeng Fu[d], Jian Zhu[a], Yanghui Liu[b], Qingyun Dou[a]*, Xingbin Yan[a]*

[a] *Department of Materials Science and Engineering, Sun Yat-sen University, Guangzhou 510275, China*

[b] *School of Materials, Sun Yat-sen University, Shenzhen, 518107, China*

[c] *Laboratory of Clean Energy Chemistry and Materials, Lanzhou Institute of Chemical Physics, Chinese Academy of Sciences, Lanzhou, 730000, PR China*

[d] *College of Materials Science and Engineering, Hunan University, Changsha, 410082, China*

- ***Corresponding Author.*** *E-mail: yanxb3@mail.sysu.edu.cn (Xingbin Yan), douqy3@mail.sysu.edu.cn (Qingyun Dou)*

Materials and Methods

Synthesis of ZIF-7

The synthesis of ZIF-7 was achieved using a conventional hydrothermal method, as described in previous studies[1]. All reagents, including zinc nitrate hexahydrate ($Zn(NO_3)_2 \cdot 6H_2O$, 98%, Fluka), benzimidazole ($C_7H_6N_2$, 98%, Aldrich), N,N-dimethylformamide (DMF) (99%, Aldrich), and methanol (CH_3OH, 99%, Aldrich), were utilized directly without additional purification.

To prepare ZIF-7, a solid mixture of $Zn(NO_3)_2 \cdot 6H_2O$ (0.8025 g, 2.7 mmol) and benzimidazole (0.2347 g, 2 mmol) was dissolved in 75 mL of DMF under ultrasonic agitation for 10 minutes. The resulting homogeneous solution was then transferred into a 125-mL Teflon-lined stainless-steel autoclave. The autoclave was sealed and heated to 130 ℃ at a rate of 5 ℃/min in an oven. After maintaining this temperature for 48 hours under autogenous pressure, the autoclave was removed from the oven and allowed to cool to room temperature. The supernatant was carefully decanted, and the product was washed with methanol. Fresh methanol was employed to replace the DMF over a period of 48 hours at room temperature. After removing the excess methanol and allowing the product to air-dry for 24 hours, white crystals were obtained.

The guest molecules within the crystals were evacuated under dynamic vacuum at 150 ℃ for 12 hours to obtain the final product.

Materials characterizations

X-ray diffraction (XRD) was performed using a powder X-ray diffractometer (D-MAX 2200 VPC, Rigaku) with Cu Kα radiation. Raman spectra were recorded employing a Reflex inVia micro-Raman spectroscope, with an excitation wavelength of 532 nm. The surface morphologies and microstructures of all the samples were analyzed using a field-emission scanning electron microscope (SEM) (SU8010) and a transmission electron microscope (TEM) (Tecnai G2 F30). XPS were achieved by X-ray photoelectron spectroscope (ESCALAB 250Xi). Pore size distribution and Brunauer-Emmett-Teller (BET) specific surface area of YP-50F activated carbon and ZIF-7 materials were measured by Autosorb-iQ. Thermal gravimetric analysis (STA449F5) was performed to determine the weight-dependent quantity of the samples at varying temperatures.

Electrochemical measurements

The working electrode slurry was prepared by mixing 80 wt.% of the active material, 10 wt.% of acetylene black, and 10 wt.% of PVDF in N-methylpyrrolidinone (NMP) solvent. The obtained mixture was applied to a nickel foam substrate (1 cm × 1 cm). The nickel foam was then dried at 80 ℃ in an oven overnight and pressed under a pressure of 10 MPa. This substrate was used for subsequent electrochemical tests. CR2032-type coin cells (supplied by Guangdong Canrd Ltd) for electrochemical measurements.The cyclic voltammetry (CV) and electrochemical impedance spectroscopy (EIS) measurements were conducted using a CHI660 electrochemical workstation from Shanghai, China. EIS was performed with an AC voltage of 10 mV amplitude over a frequency range of 0.01 Hz to 100 kHz. The YP-50F electrode was prepared by blending YP-50F carbon, acetylene black, and 10 wt.% PVDF in a mass ratio of 8:1:1 using N-methylpyrrolidinone (NMP) solvent with ethanol. The slurry resulting from the process was applied onto nickel foam and dried at 80 ℃ for 12 hours. A coin-type cell containing a 1.0 M KOH aqueous electrolyte was used for the two-electrode measurement system. The assembled cells included the ZIF-7 and YP-50F electrodes, which were positioned together and separated by a glass fiber separator. The mass of ZIF-7 was 1.60 mg, while the mass of YP-50F was 5.20 mg.

Computational details

The Vienna *ab initio* simulation package (VASP5.4.4) is utilized for all the density functional theory (DFT) calculations in this study[2]. The PBE functional is employed to simulate the exchange-correlation interactions, and the ion-electron interactions are described using the projector augmented wave (PAW) method[3, 4]. To account for vdWs interactions, the empirical DFT-D3 method is incorporated[5]. For all periodic structures, the Monkhorst-Pack grid mesh-based Brillouin zone k-points are set as 1x1x1, with a cutoff energy of 400 eV. Convergence criteria are set as 0.02 eV/Å for forces and 10^{-5} eV for energy. A cubic cell with dimensions of 20 Å x 20 Å x 20 Å is employed to represent the simplified MOF unit.

The adsorption energy ($\Delta E_{\text{adsorption}}$) is calculated using the following equation:

$$\Delta E_{\text{adsorption}} = E_{\text{total}} - E_{\text{substrate}} - E_{\text{adsorbate}} \tag{1}$$

Here, $E_{\text{substrate}}$ represents the enery of the material's surface, $E_{\text{adsorbate}}$ denotes the energy of the adsorbate, and E_{total} corresponds to the total energy of the entire adsorption system.

X-ray absorption fine structure analysis

Zn K-edge analysis was performed with Si (111) crystal monochromators at the BL11B beamlines at the Shanghai Synchrotron Radiation Facility (SSRF) (Shanghai, China). Before the analysis at the beamline, samples were pressed into thin sheets with 1 cm in diameter and sealed using Kapton tape film. The XAFS spectra were recorded at room temperature using a 4-channel Silicon Drift Detector (SDD) Bruker 5040. Zn-edge extended X-ray absorption fine structure (EXAFS) spectra were recorded in transmission mode. Negligible changes in the line-shape and peak position of Zn-edge XANES spectra were observed between two scans taken for a specific sample. The XAFS spectra of these standard samples (Zn foil and ZnO) were recorded in transmission mode. The spectra were processed and analyzed by the software codes Athena and Artemis.

Data reduction, data analysis, and EXAFS fitting were performed and analyzed with the Athena and Artemis programs of the Demeter data analysis packages[6] that utilizes the FEFF6 program[7] to fit the EXAFS data. The energy calibration of the sample was conducted through standard and Zn foil, which as a reference was simultaneously measured. A linear function was subtracted from the pre-edge region, then the edge jump was normalized using Athena software. The χ(k) data were isolated by subtracting a smooth, third-order polynomial approximating the absorption background of an isolated atom. The k^3-weighted $\chi(k)$ data were Fourier transformed after applying a HanFeng window function ($\Delta k = 1.0$). For EXAFS modeling, the global amplitude EXAFS (CN, R, σ^2 and ΔE_0) were obtained by nonlinear fitting, with least-squares refinement, of the EXAFS equation to the Fourier-transformed data in R-space, using Artemis software, EXAFS of the Zn foil are fitted and the obtained amplitude reduction factor S_0^2 value (0.805) was set in the EXAFS analysis to determine the coordination numbers (*CNs*) in sample.

For Wavelet Transform analysis, the χ(k) exported from Athena was imported into the Hama Fortran code. The parameters were listed as follow: R range, 1.0-4.0 Å, k range, 0-12.0 $Å^{-1}$; k weight, 3; and Morlet function with κ=15, σ=1 was used as the mother wavelet to provide the overall distribution.

Supplementary Note 1:

The electrodes (zeolitic imidazolate framework ZIF-7 and commercially available activated carbon YP-50F) of CAPistor were characterized by XRD, SEM, TGA, Raman, and BET analysis techniques, respectively. As shown in the **Fig. S1**, the characteristic peaks of ZIF-7 at 2θ = 7.3°, 7.8°,13.4°, 15.5°, 16.4°, 18.8°, 19.7°, and 21.3°, which are consistent with those reported in the literature[8-10], can be demonstrated that **t**he synthesized ZIF-7 material is a pure phase.

The Raman spectra assignment of ZIF-7 and benzimidazole ligands has been previously reported[11-13]. As shown in the **Fig. S2**, the peaks at position 646 cm^{-1}、646 cm^{-1} and 1583 cm^{-1} are attributed to the torsional mode of imidazole (Im) 、benzene (Bz) and Im ring bending、and C=C stretching. The peaks at positions 1002 cm^{-1} and 1019 cm^{-1} are both attributed to C-H bending in Bz. The peaks at positions 1277 cm^{-1},1294 cm^{-1},1349 cm^{-1} and 1362 cm^{-1} all correspond to C-H bending in Im and Bz. Raman measurements further confirm the successful synthesis of ZIF-7 crystals.

The morphology and structure of the material were investigated through SEM characterization. The results, shown in **Fig. S3**, reveal that ZIF-7 is composed of irregular flake-like crystals with particle sizes ranging from 6 μm to 10 μm.

The molecular formula of ZIF-7 is Zn $(PhIM)_2(H_2O)_3$. According to thermal gravimetric analysis (**Fig. S4**), the ZIF-7 molecule undergoes dehydration during weightlessness, resulting in the breaking of the C-Zn-N, C-C, and C=N bonds in the molecule. This process leaves only carbon, which is then carbonized[14, 15].

Gas adsorption measurements were conducted on the ZIF-7 electrode to investigate its pore properties, surface area, and pore size distribution. The material exhibited a typical type IV adsorption and desorption isotherm **(Fig. S5a**) with a significant adsorption hysteresis loop in the intermediate section, indicating a mesoporous main pore structure. The pore size distribution pattern in **Fig. S5b** indicates that the average pore size of ZIF-7 ranges from 2 to 10 nm.

SEM images (**Fig. S6a** and **Fig. S6b**) show that YP-50F particles are irregularly square-shaped and have a size of approximately 10 μm. Raman spectra (**Fig. S7**) also exhibit two significant characteristic peaks of the carbon material: the D-band and the G-band peak. YP-50F displayed a type I isotherm (**Fig. S8a**), with a rapid increase in adsorption at lower relative pressures and a saturation value after reaching a certain relative pressure, representing the phenomenon of microporous infilling. Furthermore, the pore size distribution (**Fig. S8b**) indicates that the pore size of YP-50F is concentrated within the range of 0.55~2.5 nm.

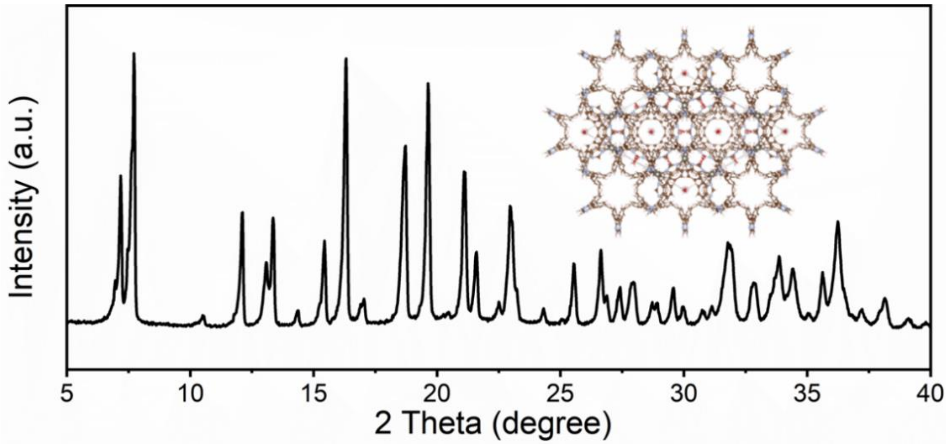

Fig. S1. XRD pattern of ZIF-7.

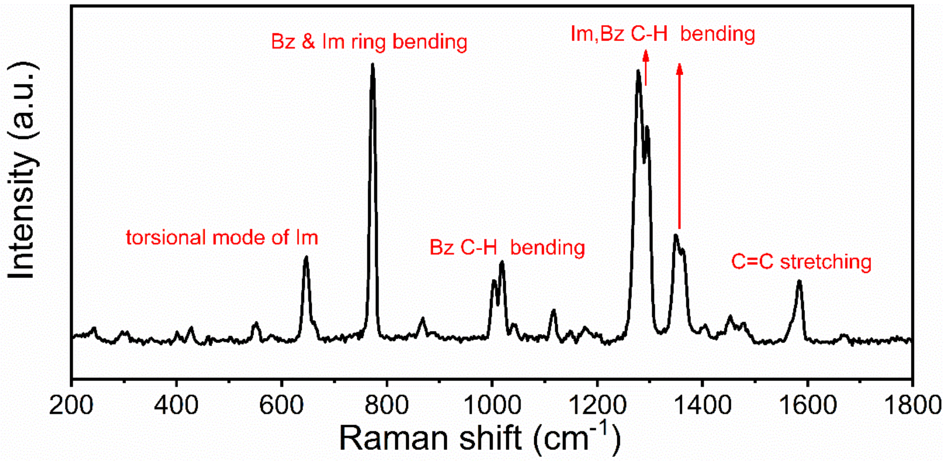

Fig. S2. The Raman spectrum of ZIF-7.

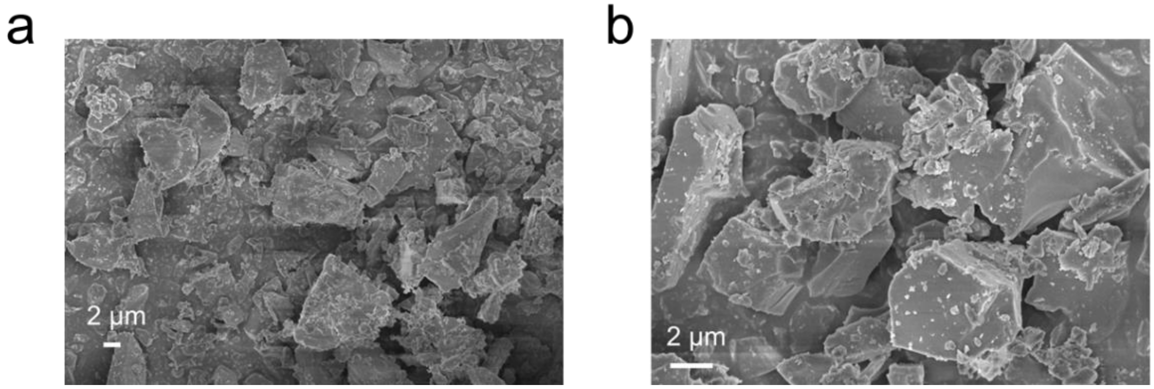

Fig. S3. (a) Low magnification SEM image of ZIF-7. Scale bar: 2 μm. (b) High magnification SEM image of ZIF-7. Scale bar: 2 μm.

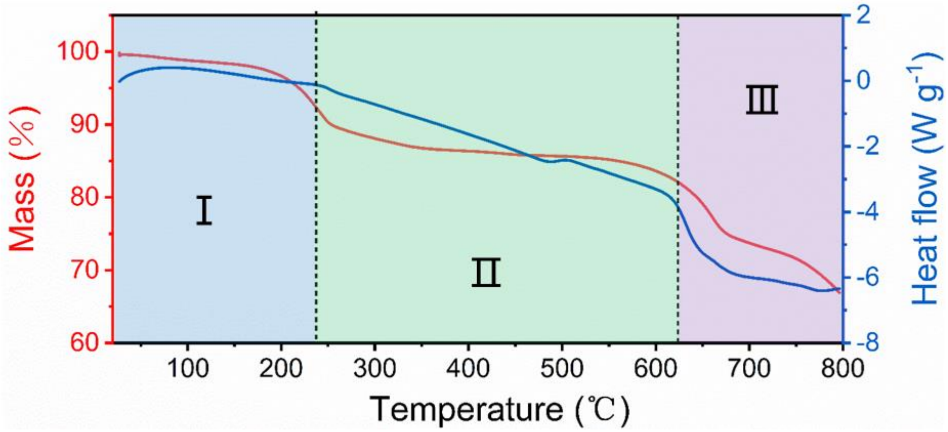

Fig. S4. Thermogravimetry curves of ZIF-7.

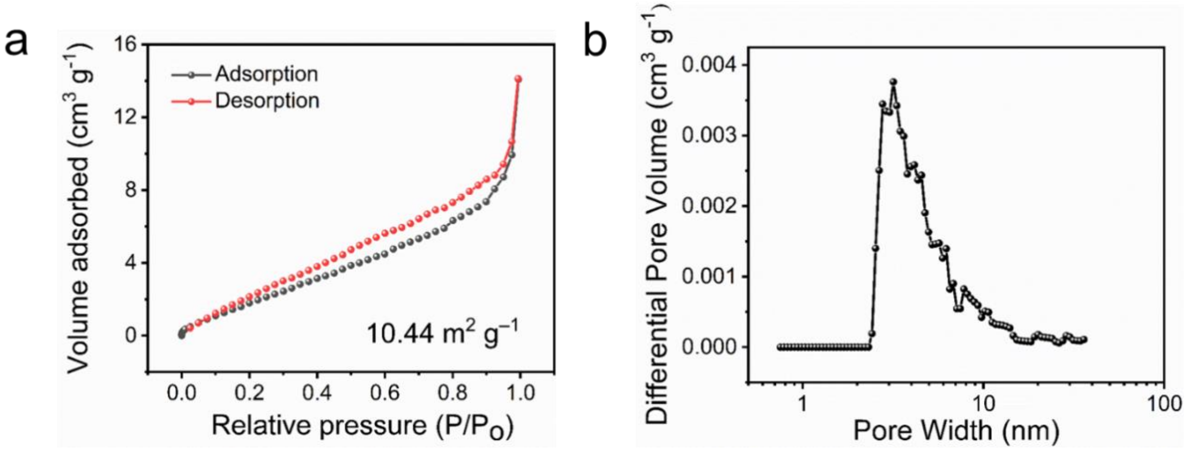

Fig. S5. (a) N_2 adsorption-desorption isotherm of ZIF-7. (b) Pore size distribution of ZIF-7.

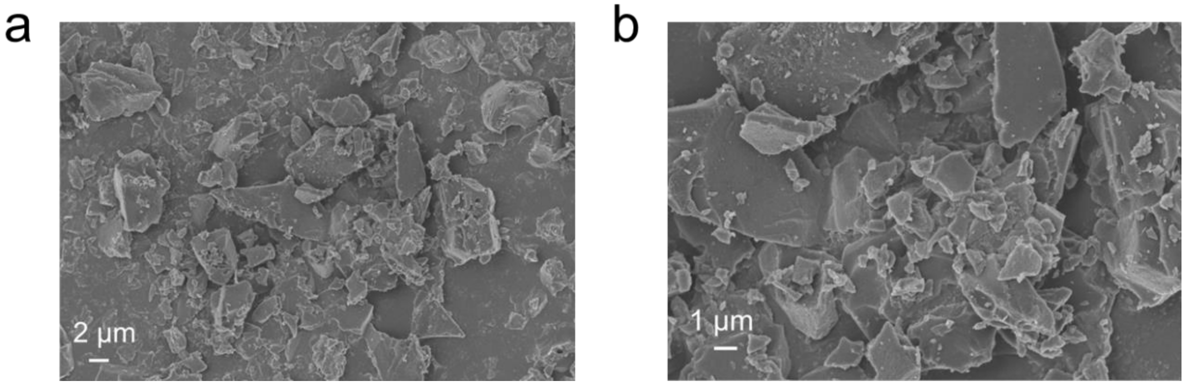

Fig. S6. (a) Low magnification SEM image of YP-50F. Scale bar: 2 μm. (b) High magnification SEM image of YP-50F. Scale bar: 1 μm.

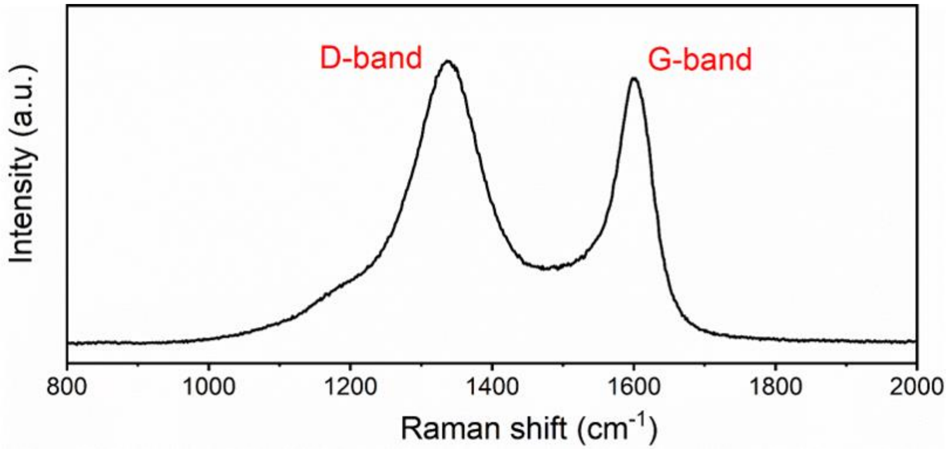

Fig. S7. The Raman spectrum of YP-50F.

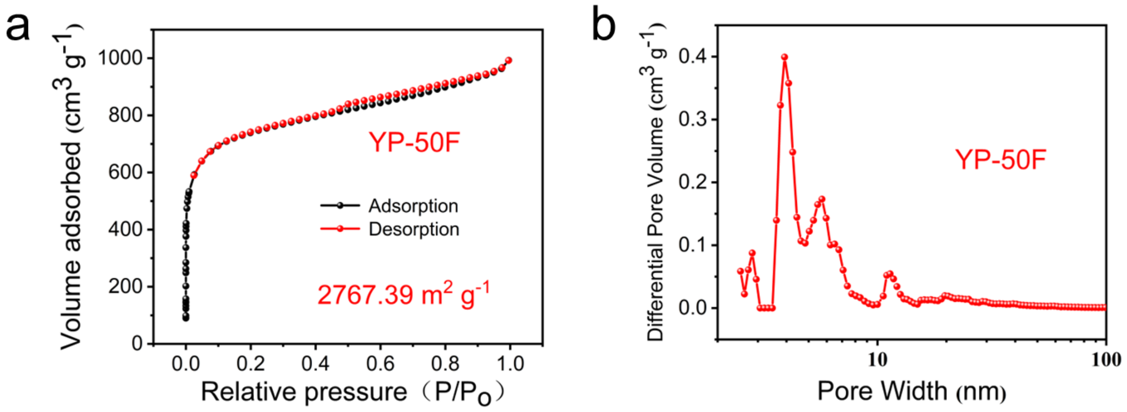

Fig. S8. (a) N_2 adsorption-desorption isotherm of YP-50F. (b) Pore size distribution of YP-50F.

Supplementary Note 2:

Fig. S9 shows the fabricated CAPistor by sequentially assembling the carbon material electrode, glass fiber (separator), and metal-organic framework electrode in the order of adding aqueous alkaline electrolyte and encapsulation. The response of the current to a linear triangular wave voltage is non-linear, as depicted in **Fig. S10**.

The feasible temperature range of the CAPistor was evaluated. **Fig. S12a** shows that the device's I-U curve exhibits good resistive switching characteristics from 20 ℃ to 60 ℃. Electrochemical impedance spectroscopy (EIS) measurements in **Fig. S12b** indicate that the slope in the low frequency range increases with temperature, implying an acceleration of ion transfer in the CAPistor.

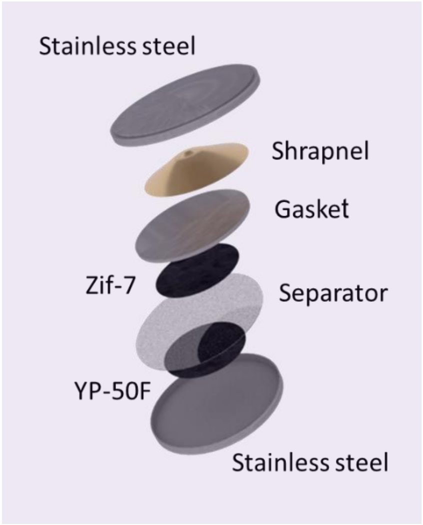

Fig. S9. Schematic illustration of assembling supercapacitor–memristor.

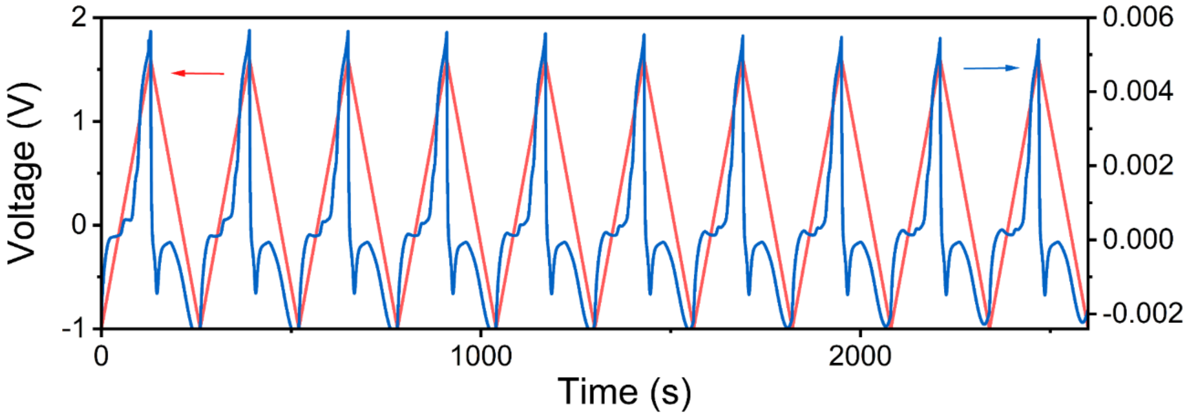

Fig. S10.Trend of voltage versus current at different times converted from CV curves.

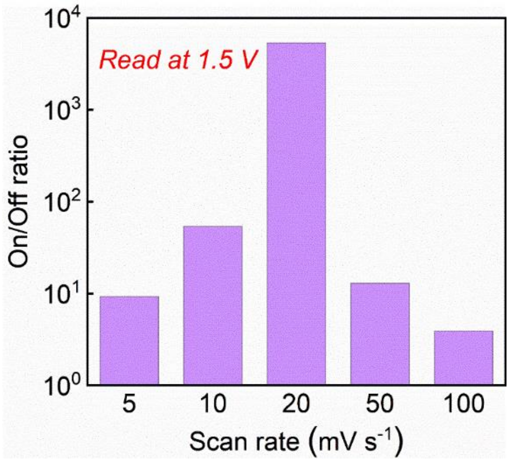

Fig. S11. The ON/OFF ratio read at 1.5 V of CAPistor at different scan rates.

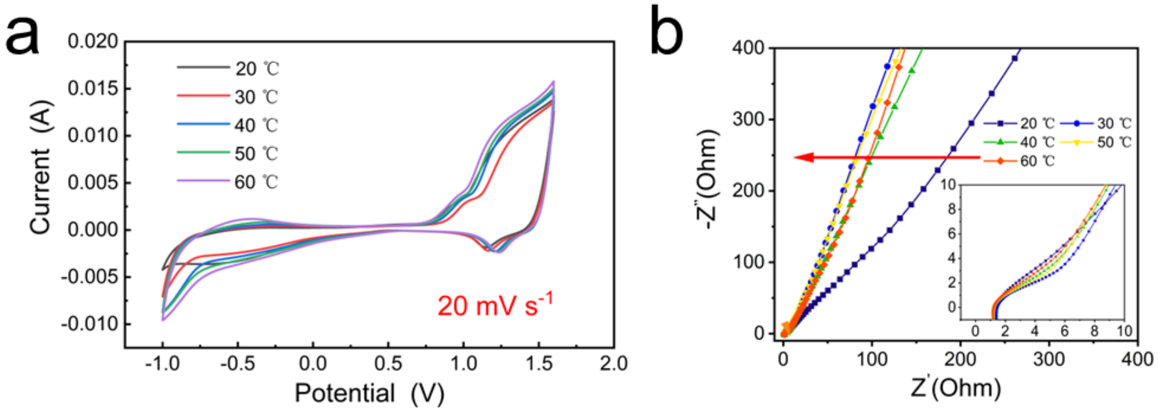

Fig. S12. Performance evaluation of CAPistor at different temperatures. (a) CV curves of CAPistor at different temperatures. (b) Nyquist plots of CAPistor at different temperatures.

Supplementary Note 3:

As described in the main text, the CV curve was divided into four parts. The **Fig. S13** shows that S_2 only occupies a small portion of the area of S_1 at different scan rates (approximately 10% to 20%), while S_3 occupies a larger portion of the area of S_1 (approximately between 60% and 80%). The sum of S_2 and S_3 accounts for approximately 90% of the area of S_1. The coulombic efficiency of the device is extremely low (the ratio of S_2 to S_1 equals approximately 20%) when calculated from a conventional supercapacitor perspective. The discharge specific capacity, calculated from the GCD curve, is much lower than the charge specific capacity (**Fig. S14a**). The coulombic efficiency is 21% at a current density of 0.5 A g^{-1} (**Fig. S14b**), which is consistent with the results obtained from the CV curve. If the coulombic efficiency is calculated from the full voltage range, the device achieves normal levels. The ratio of the sum of S_2 and S_3 to S_1 is approximately equal to 90%.

The gravimetric capacity was calculated via the following equation (2)[16-18]:

$$C = \frac{I\Delta t}{m\Delta V} \tag{2}$$

Where C is the specific capacitance (F g^{-1}) based on GCD curves, I represent the discharge current (mA), Δt represent the discharge time in seconds (s), ΔV represent the voltage variation upon discharging and charging (V), and m represent the mass of the active material (mg) (The mass of ZIF-7 was 1.6 mg).

Fig. S15 shows that the area of the CV curve of the CAPistor decreases significantly in the first five cycles, especially at high scan rates. This may be due to the fact that the OH^- ions migrating into the MOF pores during charging cannot be properly detached, and thus the active site decreases, leading to a decrease in capacitance during the next cycle. As shown in **Fig. S16**, the positive potential region for scanning in the full voltage interval (−1-1.6V) is always larger than the area of the CV curve for scanning only in the positive voltage interval (0-1.6V) at different scanning rates. This phenomenon suggests that negative scanning has a restoring effect on the CAPistor by removing excess OH^- anion from the ZIF-7 pore, thereby increasing the active sites. Long-cycle testing showed that the capacity of the CAPistor decayed to 95.8% after 100 cycles and remained at a high level. In addition, the coulombic efficiency increased from 22.1% in the first cycle to 28.8% in 100 cycles and remained at a lower level.

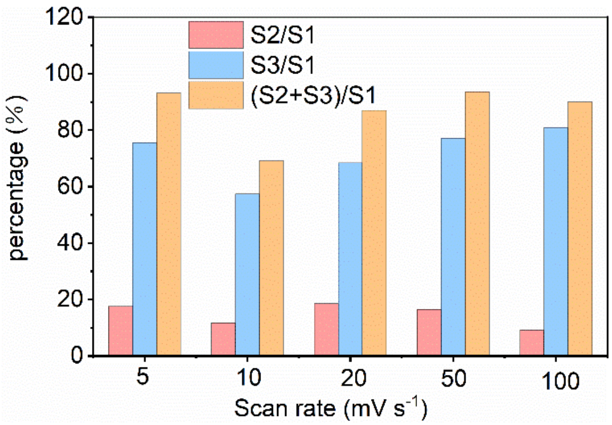

Fig. S13. The percentage of different areas in the CV curves.

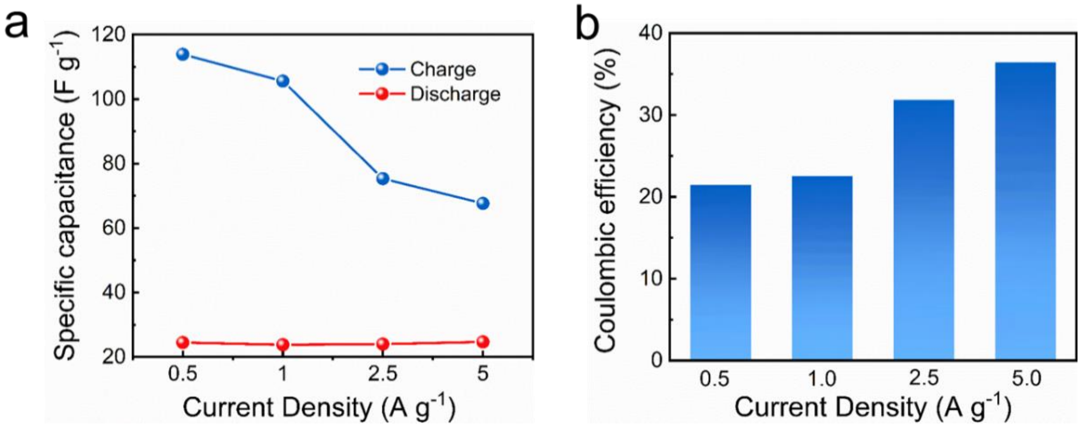

Fig. S14. (a) Specific capacity of the CAPistor at different current densities calculated from the charging and discharging curves. (b) The corresponding coulombic efficiency at different current densities.

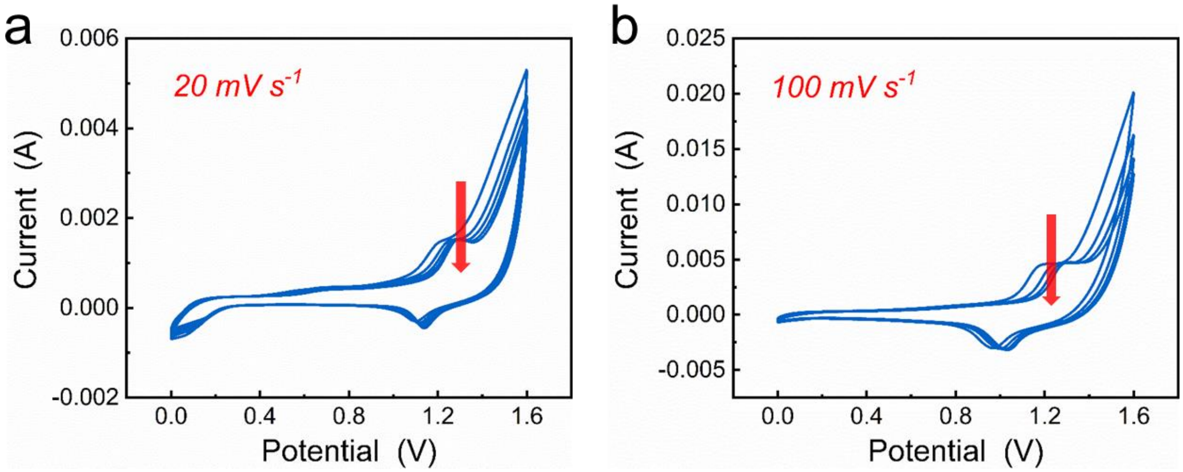

Fig. S15. (a) CV curves at 20 mV s^{-1} for the first 5 cycles of CAPistor with cycling only in the positive voltage range. (b) CV curves at 100 mV s^{-1} for the first 5 cycles of CAPistor with cycling only in the positive voltage range.

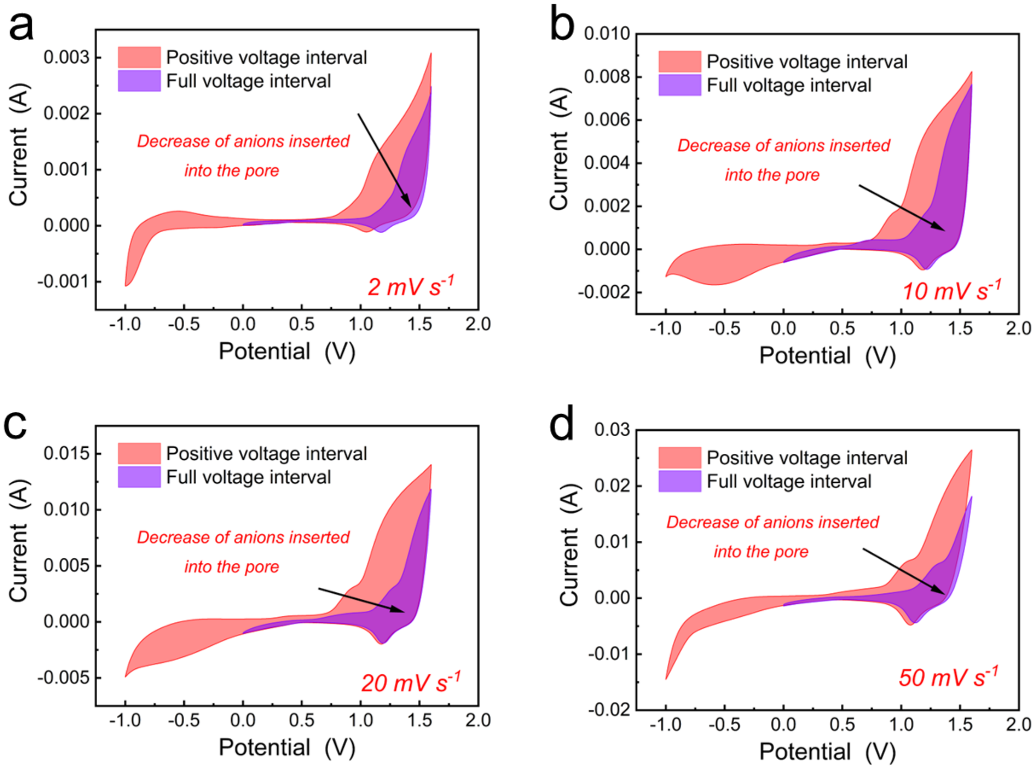

Fig. S16. (a-d) Comparison of CV curves at different scan rates (2 mV s^{-1}、10 mV s^{-1}、20 mV s^{-1} and 50 mV s^{-1}) for positive and full voltage intervals.

Supplementary Note 4:

To further analyze the transport kinetics of OH^- anions in ZIF-7 pores, we obtained more information by studying the CV curves of CAPistor at different voltages. From a positive voltage window of 1.0 V to 1.6 V, CAPistor exhibits a squeezing hysteresis loop at different scan rates (**Fig. S17**). In particularly, we observe a significant current response in the first and third parts of the curve, which exhibit typical ionic memristor characteristics.

The shape of the CV curves changes as the scan rate increases (**Fig. S17** and **Fig. S18a-g**). This can be attributed to the fact that at high scan rates, OH^- cannot insert and exsert from the MOF pore in time, which weakens the hysteresis effect. At a scan rate of 100 mV s^{-1}, the area of the second part S_2 gradually decreases while the area of the third part S_3 gradually increases as the voltage window increases from 1.0 V to 1.6 V (**Fig. S18h-i**). Conversely, the area of the second part S_2 gradually increases with the increase of the voltage window. This phenomenon may be attributed to the increase in the amount of OH^- anions inserted into the pores of ZIF-7 as the positive potential window gradually increases, while the amount of extracted OH^- anions from the second part (decreasing positive potential) remains constant. Additionally, the amount of remaining delocalized OH^- in the third part (increasing positive potential) increases. In simpler terms, as the positive potential window increases, more OH^- ions are inserted into the pore channel, resulting in a more pronounced squeezing hysteresis characteristic in the CV curve. To ensure a detailed study, we selected a voltage window of −1 V to 1.6 V. The squeezing hysteresis characteristic of the CV curve becomes more obvious as the positive potential window increases. To ensure a detailed study, we selected a voltage window of −1 V to 1.6 V.

To ensure the reproducibility of the data, we assembled four different supercapacitor memristors. As shown in the **Fig. S21**, each of these devices exhibits a nonlinear current-voltage (I-V) characteristic when a time-varying voltage is applied. This squeezing loop that occurs under periodic forcing is a typical feature of the memristor.

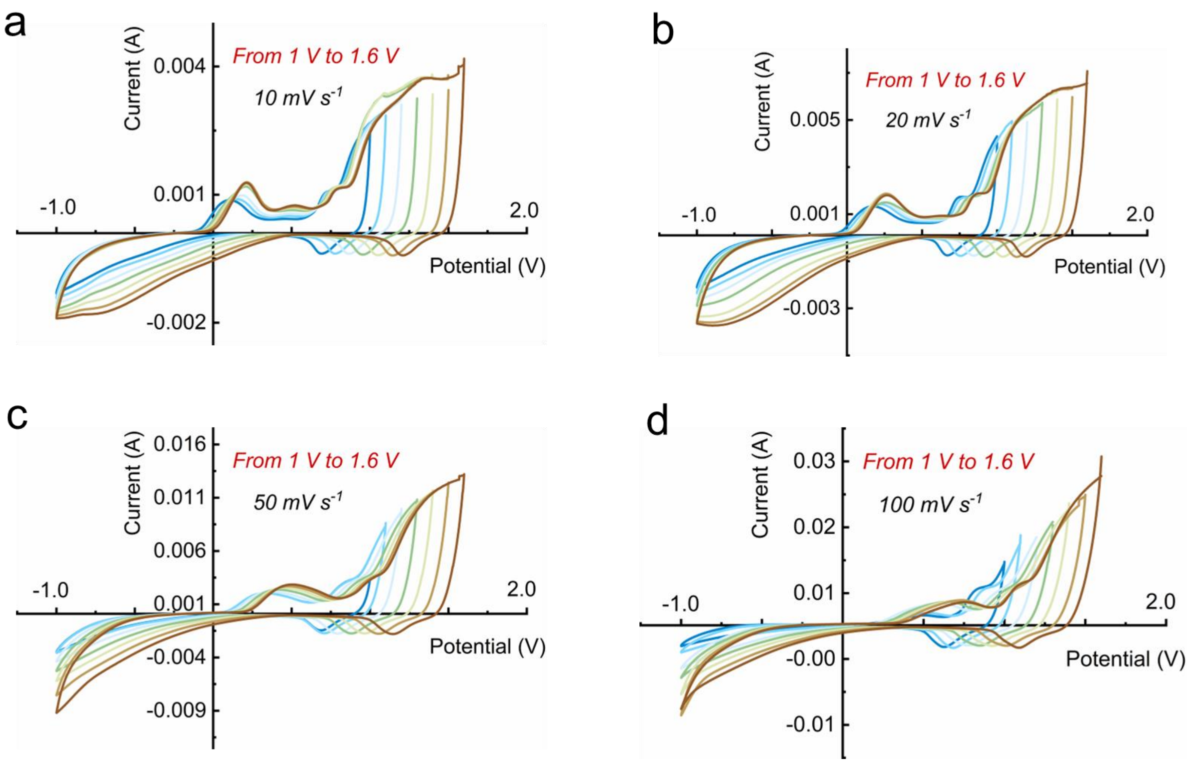

Fig. S17. CV curves for different voltage windows at (a) 10 mV s^{-1}; (b) 20 mV s^{-1}; (c) 50 mV s^{-1}; (d) 100 mV s^{-1}.

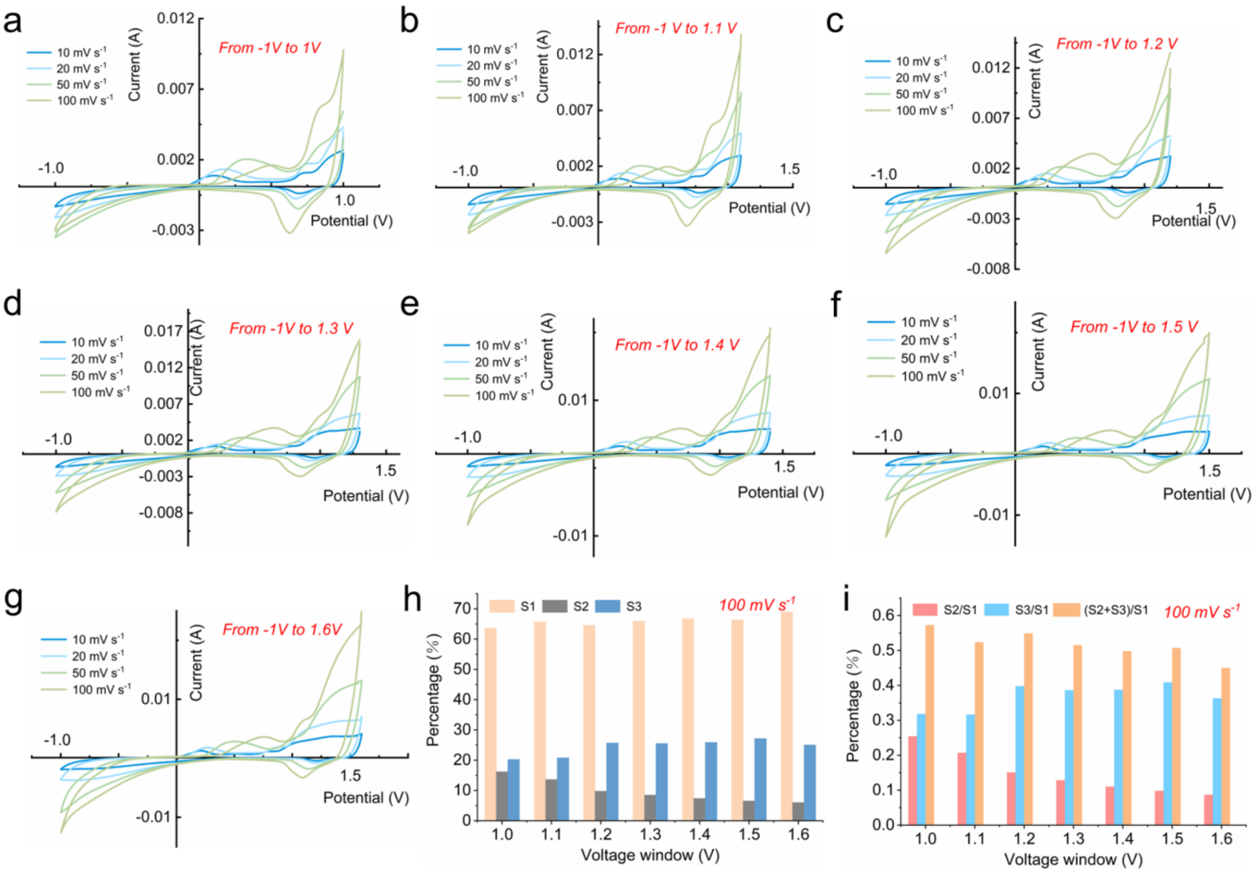

Fig. S18. CV curves at different scanning rates for different voltage windows (a) from −1 V to 1 V；(b) from −1 V to 1.1 V；(c) from −1 V to 1.2 V；(d) from −1 V to 1.3 V；(e) from −1 V to 1.4 V；(f) from −1 V to 1.5 V；(g) from −1 V to 1.6 V. (h-i) Percentage of area in different quadrants of the CV curve at 100 mV s^{-1} under different voltage windows.

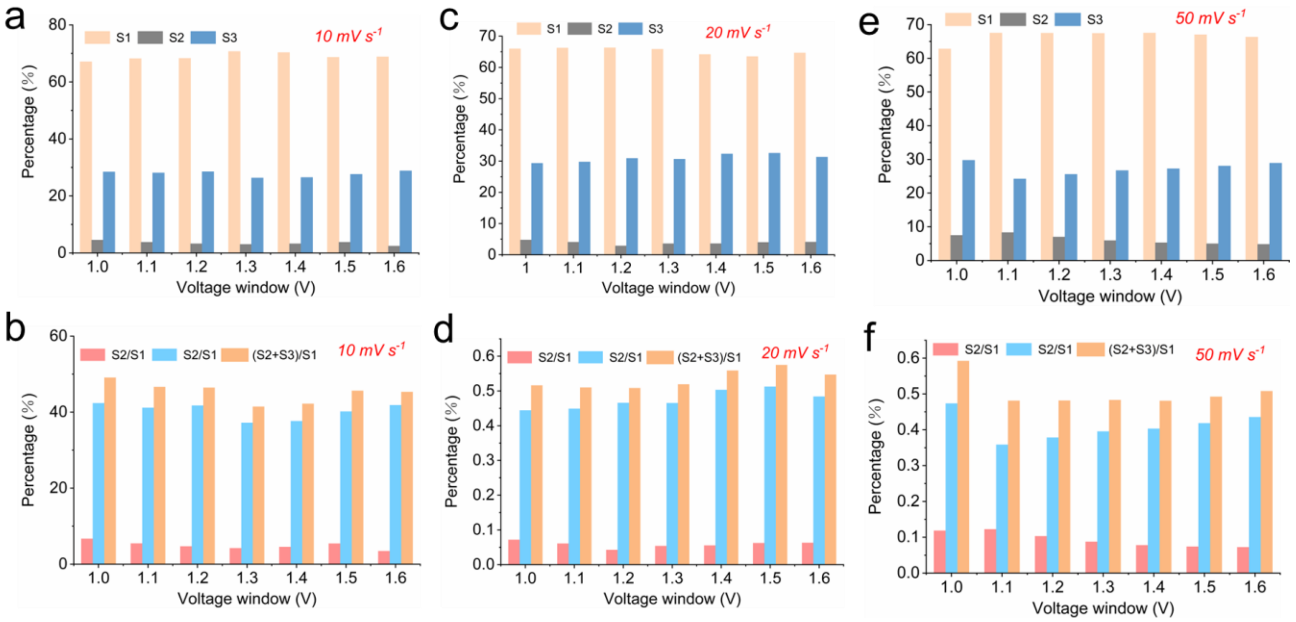

Fig. S19. Percentage of area in different quadrants of the CV curve under different voltage windows at (a-b) 10 mV s^{-1}; (c-d) 20 mV s^{-1}; (e-f) 50 mV s^{-1}.

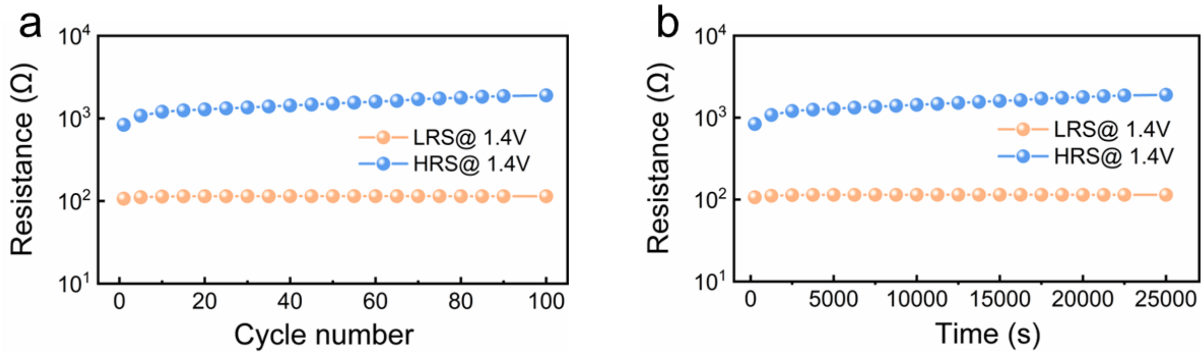

Fig. S20. (a) Resistance values read at 1.4 V in HRS and LRS for different number of cycles. (b) Resistance values read at 1.4 V in HRS and LRS for different time during the cycle.

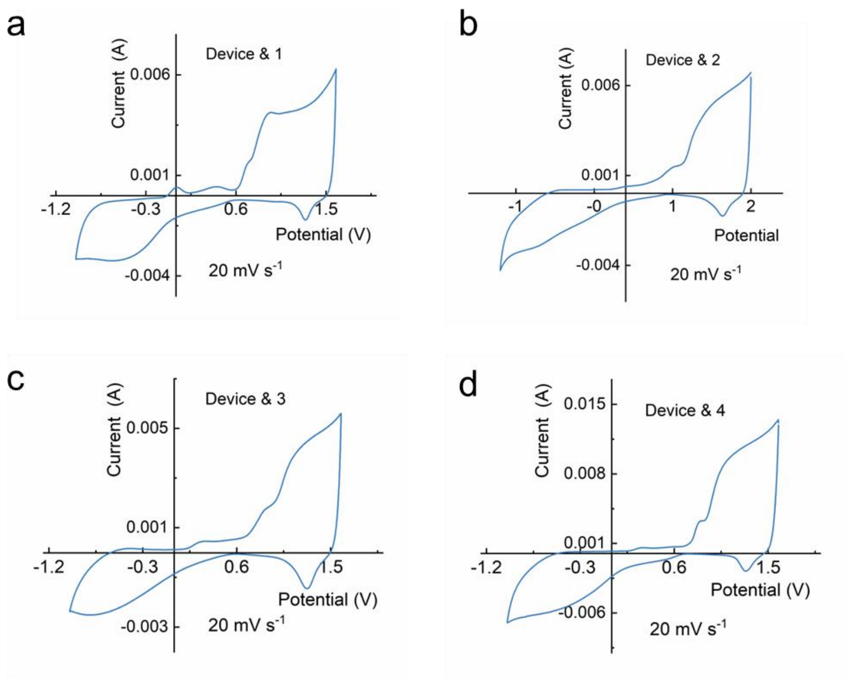

Fig. S21.Repeatability of CV curves for four assembled different supercapacitor memristors.

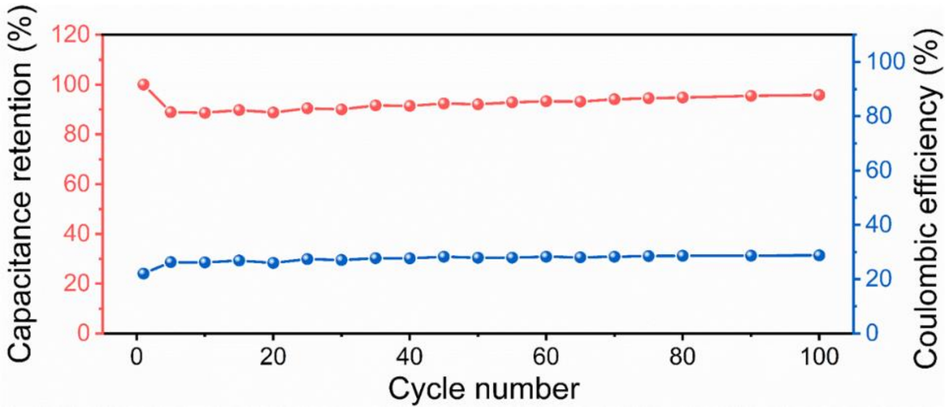

Fig. S22. Capacitance retention and coulombic efficiency at 100 mV s^{-1} during 100 cycles.

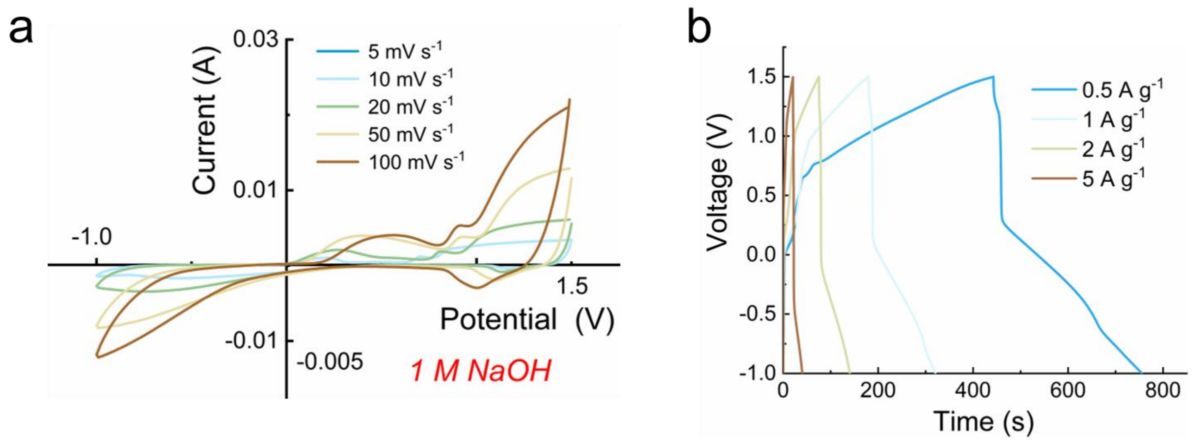

Fig. S23. (a) CV curves of CAPistor constructed with NaOH solution. (b) The corresponding GCD curves.

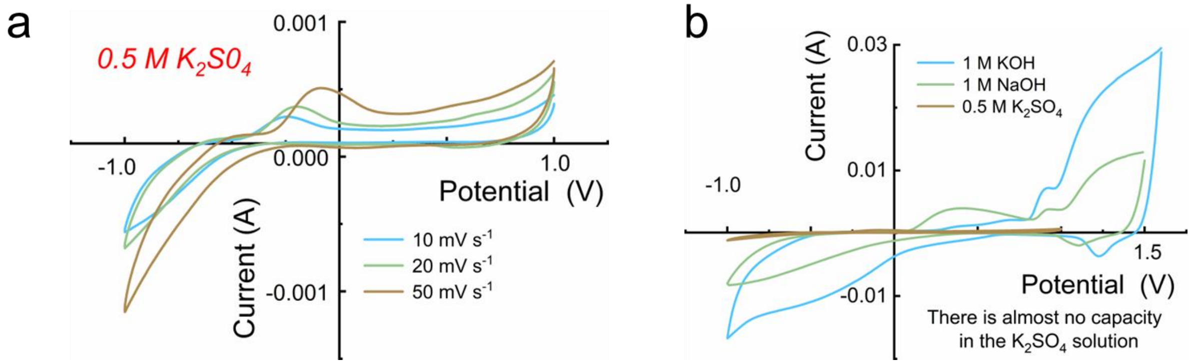

Fig. S24. (a) CV curves of CAPistor constructed with K_2SO_4 solution. (b) Comparison of CV curves of devices constructed in three different electrolytes.

Supplementary Note 5:

For a typical porous electrode, its equivalent series resistance (R_{ESR}) consists of three components: intrinsic ohmic resistance (R_{Ω}), interfacial charge transfer resistance (R_{ct}), and Warburg diffusion resistance (R_w). R_w arises from the resistance of ion diffusion, and the ion diffusion coefficient can be determined adopting the following equation[19]:

$$D = \frac{R^2T^2}{2n^4F^4A^2C^2\sigma^2} \tag{3}$$

Here, D (cm^2 s^{-1}) represents the ion diffusion coefficient, R (J mol^{-1} K^{-1}) is the gas constant, T (K) is the absolute temperature, n is the number of electrons involved in the redox reaction, A (cm^2) is the geometric area of the electrode, F (C mol^{-1}) is the Faraday constant, C (mol cm^{-3}) is the molar concentration of ions, and σ (Ω $s^{-1/2}$) is the Warburg coefficient.

Furthermore, the Warburg coefficient σ of CAPistor under different voltages (**Fig. S25**) can be determined by plotting Z' against $\omega^{-1/2}$ based on the equation[20]

$$Z' = R_{\Omega} + R_{ct} + \sigma\omega^{-1/2} \tag{4}$$

As can be seen from Eq. 3, the diffusion coefficient (D) is inversely proportional to the Warburg diffusion coefficients (σ). This means that the larger the Warburg diffusion coefficients, the smaller the diffusion coefficient and the slower the diffusion. Conversely, the smaller the Warburg diffusion coefficients, the larger the diffusion coefficient and the faster the diffusion.

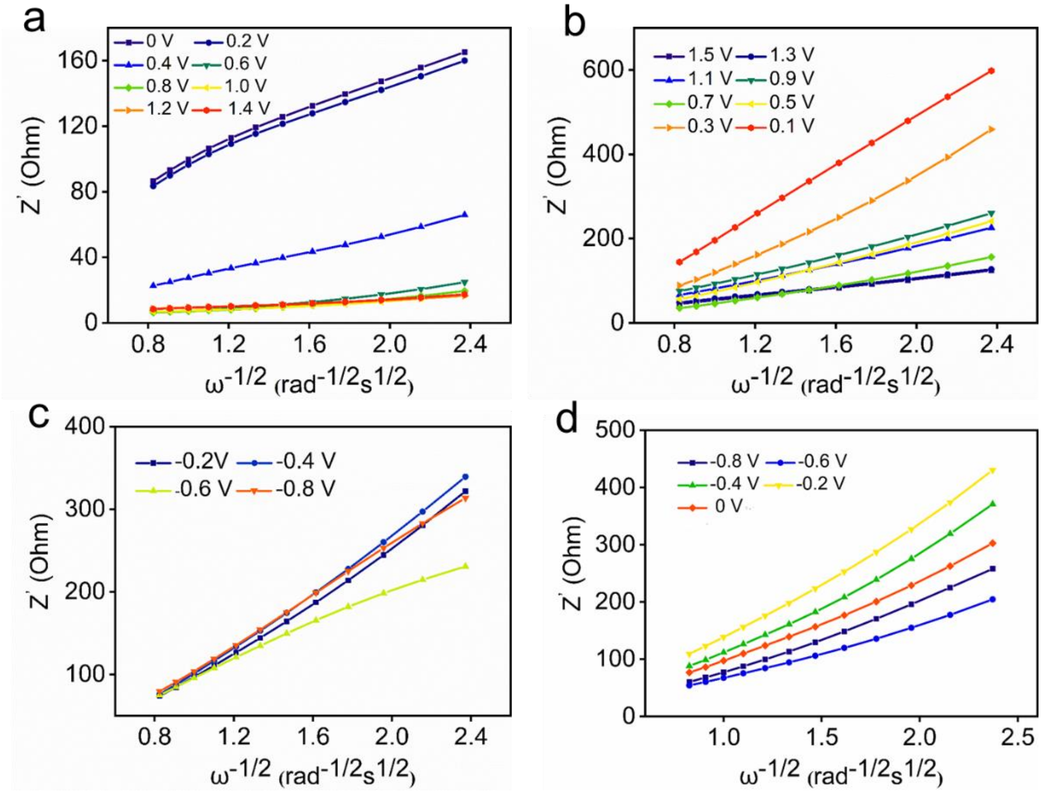

Fig. S25. Z'-$\omega^{-1/2}$ relationship acquired from EIS experiments at different voltages for CAPistor. (a) From 0 V to 1.5 V. (b) From 1.5 V to 0 V. (c) From 0 V to −1 V. (d) From −1 V to 0 V.

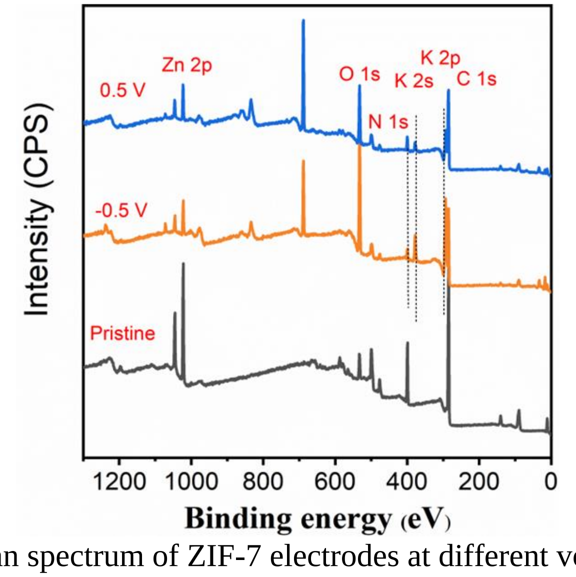

Fig. S26. XPS survey scan spectrum of ZIF-7 electrodes at different voltage bias.

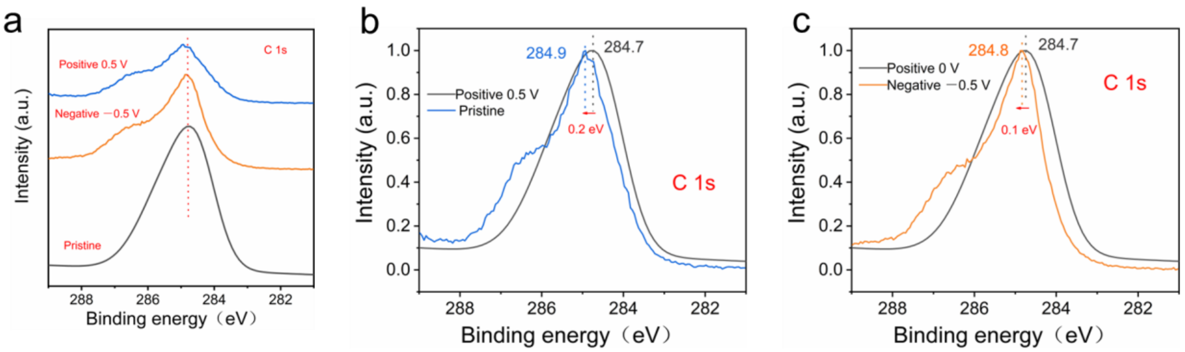

Fig. S27. (a) C 1s XPS spectra of ZIF-7 electrodes at different voltage bias. (b) Comparison of XPS spectra of C 1s of the pristine ZIF-7 electrode and the electrode subjected to a positive bias of 0.5 V. (c) Comparison of XPS spectra of C 1s of the pristine ZIF-7 electrode and the electrode subjected to a negative voltage bias of 0.5 V.

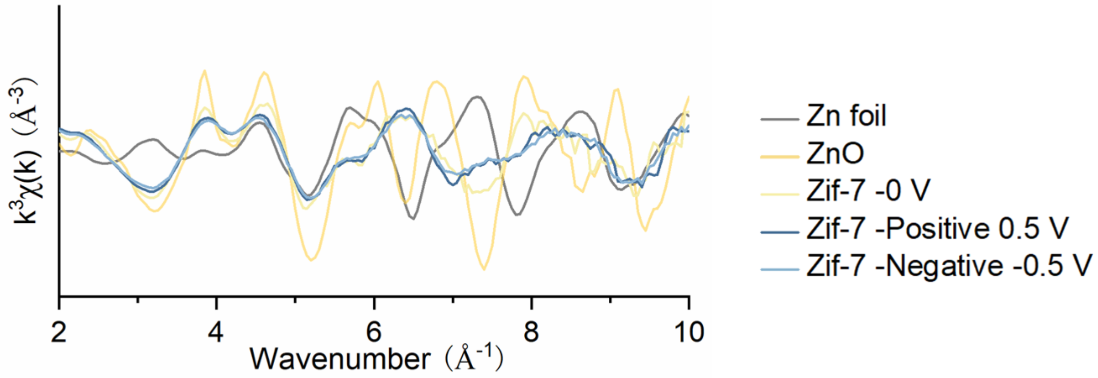

Fig. S28. EXAFS $k^3\chi(k)$ spectra at Zn K edge of ZIF-7 electrode at different potentials, Zn foil and ZnO.

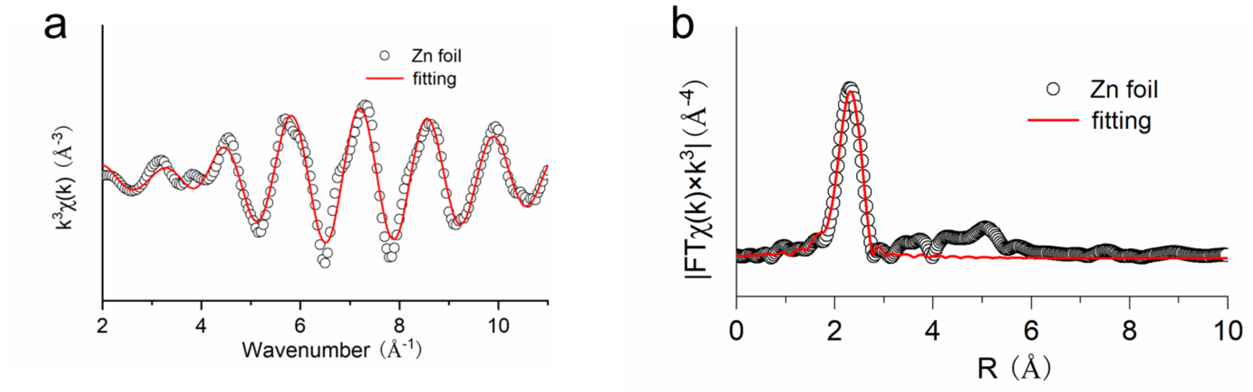

Fig. S29. (a) EXAFS $k^3\chi(k)$ spectra at Zn K edge of Zn foil. (b) EXAFS $k^3\chi(R)$ spectra and fitting results at Zn K edge of Zn foil.

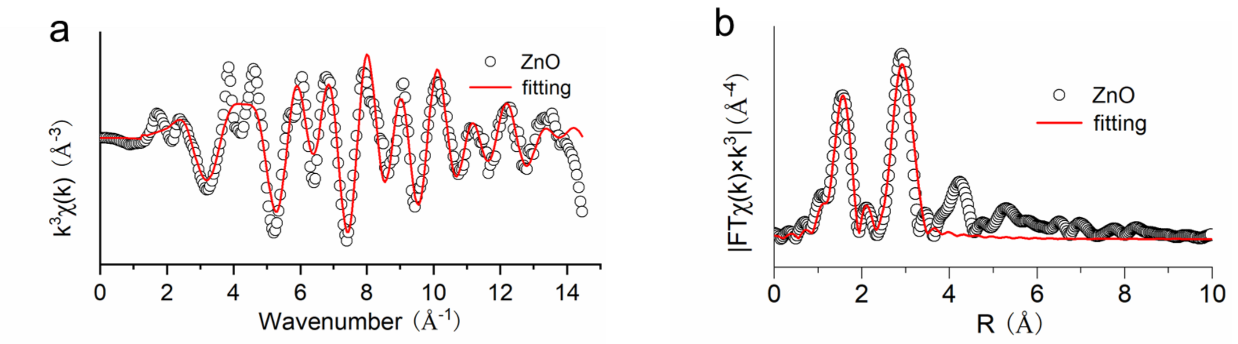

Fig. S30. (a) EXAFS $k^3\chi(k)$ spectra at Zn K edge of ZnO. (b) EXAFS $k^3\chi(R)$ spectra and fitting results at Zn K edge of ZnO.

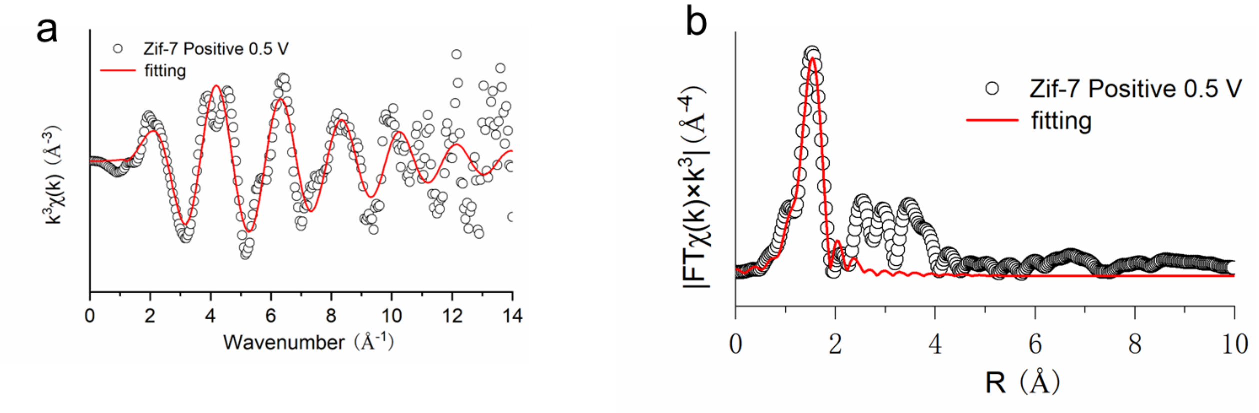

Fig. S31. (a) EXAFS $k^3\chi(k)$ spectra at Zn K edge of ZIF-7 electrodes applying a positive 0.5V voltage. (b) EXAFS $k^3\chi(R)$ spectra and fitting results at Zn K edge of ZIF-7 electrodes applying a positive 0.5V voltage.

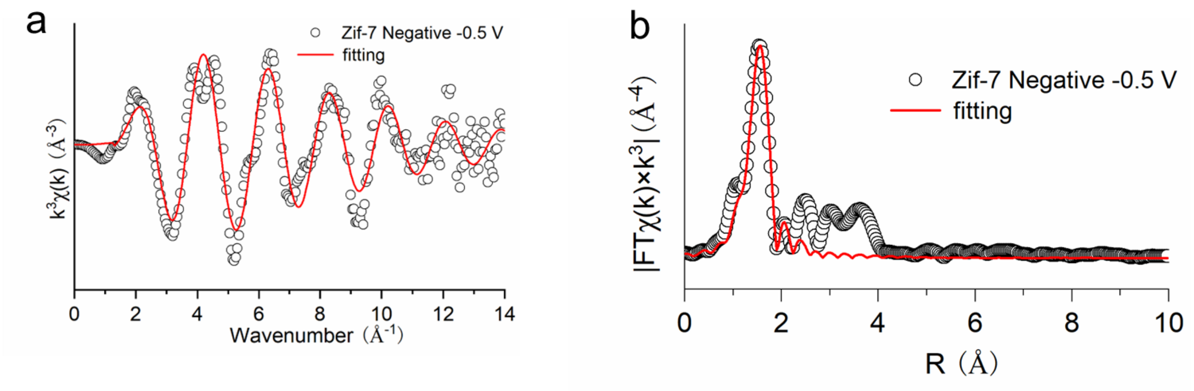

Fig. S32. (a) EXAFS $k^3\chi(k)$ spectra at Zn K edge of ZIF-7 electrodes applying a negative −0.5 V voltage. (b) EXAFS $k^3\chi(R)$ spectra and fitting results at Zn K edge of ZIF-7 electrodes applying a negative −0.5 V voltage.

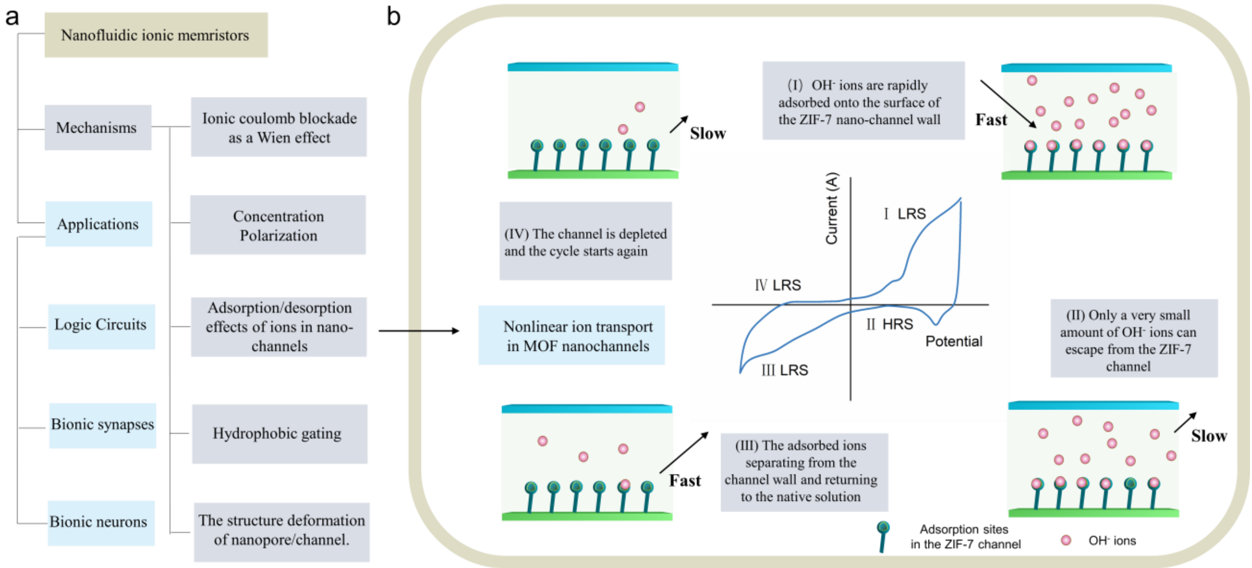

Fig. S33. (a) Classification of mechanisms and applications of nanofluidic ionic memristors. (b) Schematic representation of nonlinear ion transport in supercapacitor memristors attributed to adsorption/desorption effects of ions in nanochannels.

Supplementary Note 6:

The second to fourth running cycles (from **Fig. S34** to **Fig. S40**) show the same current changes as the first cycle in the main text. Similarly, the corresponding current for positive scanning is much higher than for negative scanning. Following the same trend as the first cycle, the subsequent cycles likewise exhibited gradient conductivity changes when positive or negative unidirectional voltage scans were applied.

The correlation between scan rate and current drop value was further investigated. As shown in **Fig. S40c**, when the scan rate is 50 mV s^{-1}, the current value read at 1.6 V decreases from 0.022 A at the first cycle to 0.017 A at the fifth cycle. Notably, when the scan rate increases to 200 mV s^{-1}, the current value decreases from 0.056 A at the first cycle to 0.029 A at the 5th cycle. It is worth noting that the current decays faster when the unidirectional scanning rate is increased.

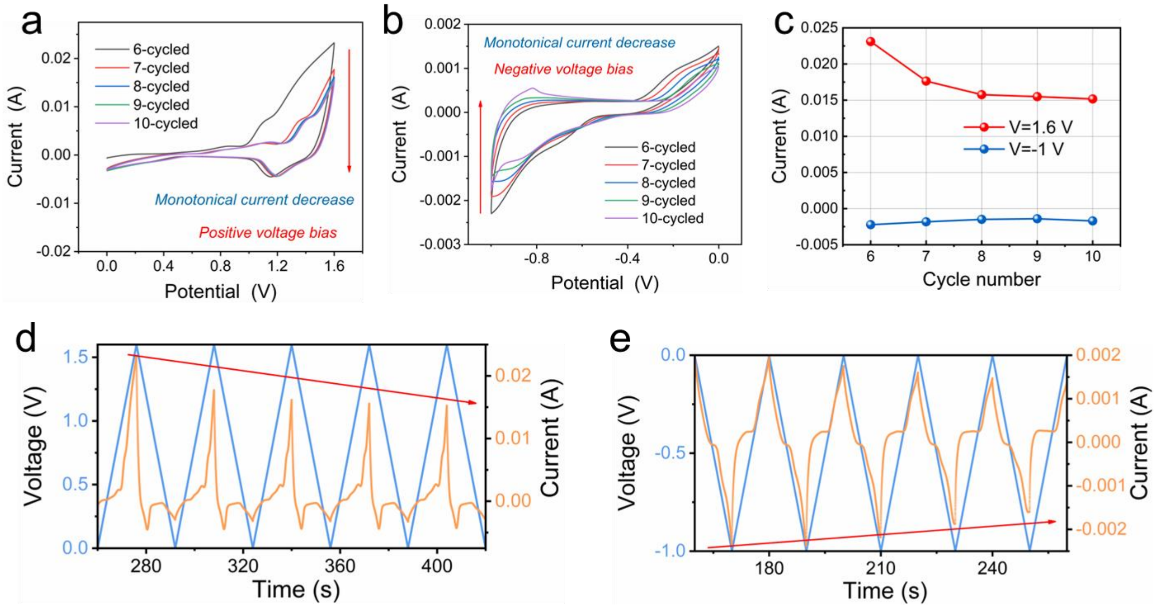

Fig. S34. I-U characteristics of the CAPistor from 6th to 10th cycles. (a, b) The independent I-V characteristics of CAPistor at positive voltage bias (0→1.6→0 V) and negative voltage bias (0→−1.0→0 V), respectively. (c) Current variations at 1.6 or −1.0 V obtained from (a) and (b) are plotted as a function of cycle numbers. (d, e) Evolution of the current (orange) under positive and negative voltage pulses of constant polarity (blue).

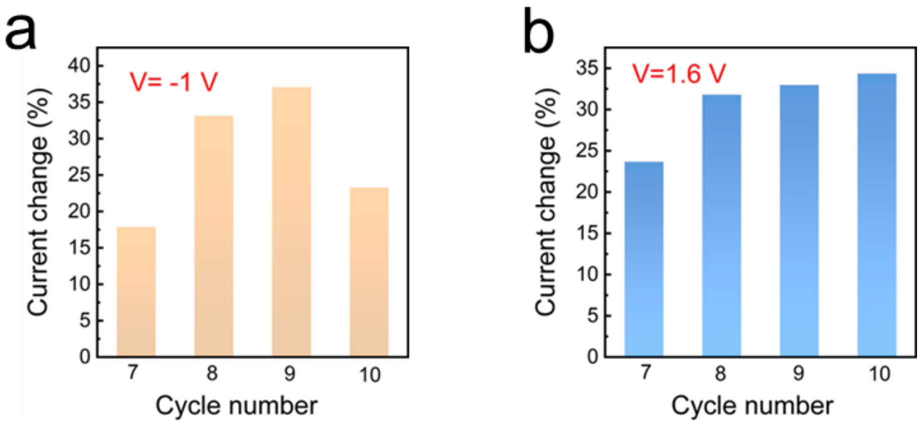

Fig. S35. (a) Current change from 6th to 10th cycles under continuous negative voltage stimulus (V= −1 V). (b) Current change from 6th to 10th cycles under continuous positive voltage stimulus (V= 1.6 V).

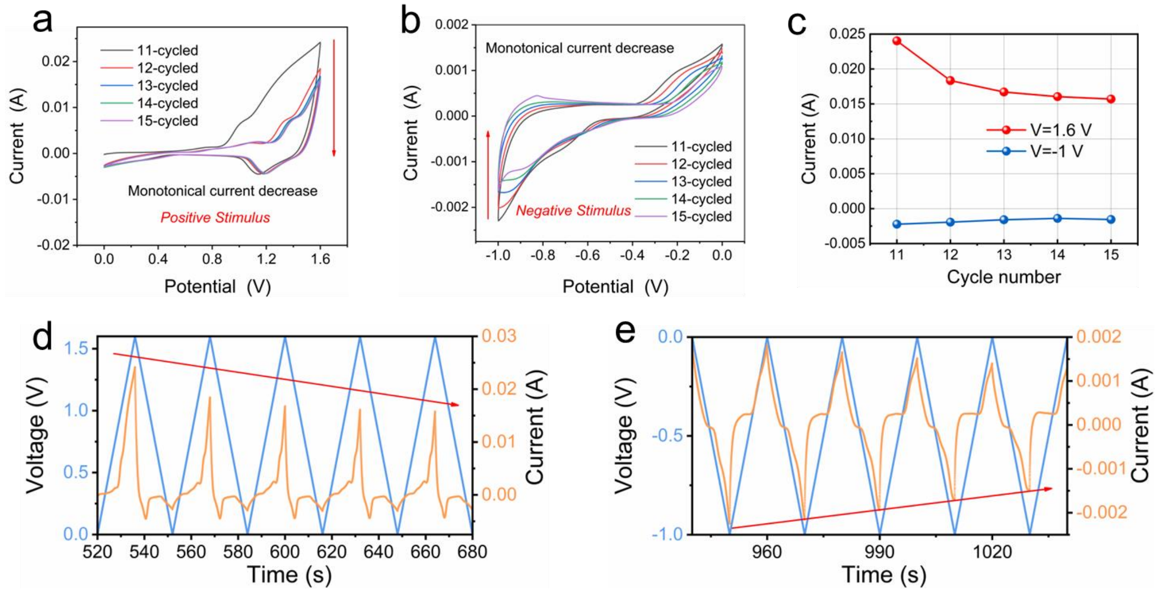

Fig. S36. I-U characteristics of the CAPistor from 11th to 15th cycles. (a, b) The independent I-V characteristics of CAPistor at positive voltage bias (0→1.6→0 V) and negative voltage bias (0→−1.0→0 V), respectively. (c) Current variations at 1.6 or −1.0 V obtained from (a) and (b) are plotted as a function of cycle numbers. (d, e) Evolution of the current (orange) under positive and negative voltage pulses of constant polarity (blue).

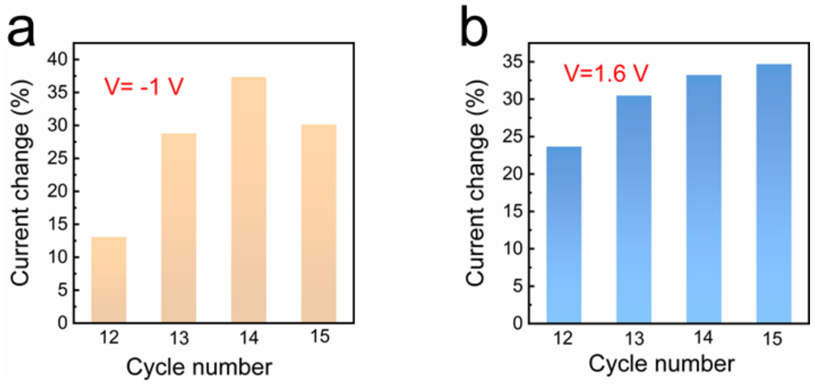

Fig. S37. (a) Current change from 11th to 15th cycles under continuous negative voltage stimulus (V= −1 V). (b) Current change from 11th to 15th cycles under continuous positive voltage stimulus (V= 1.6V).

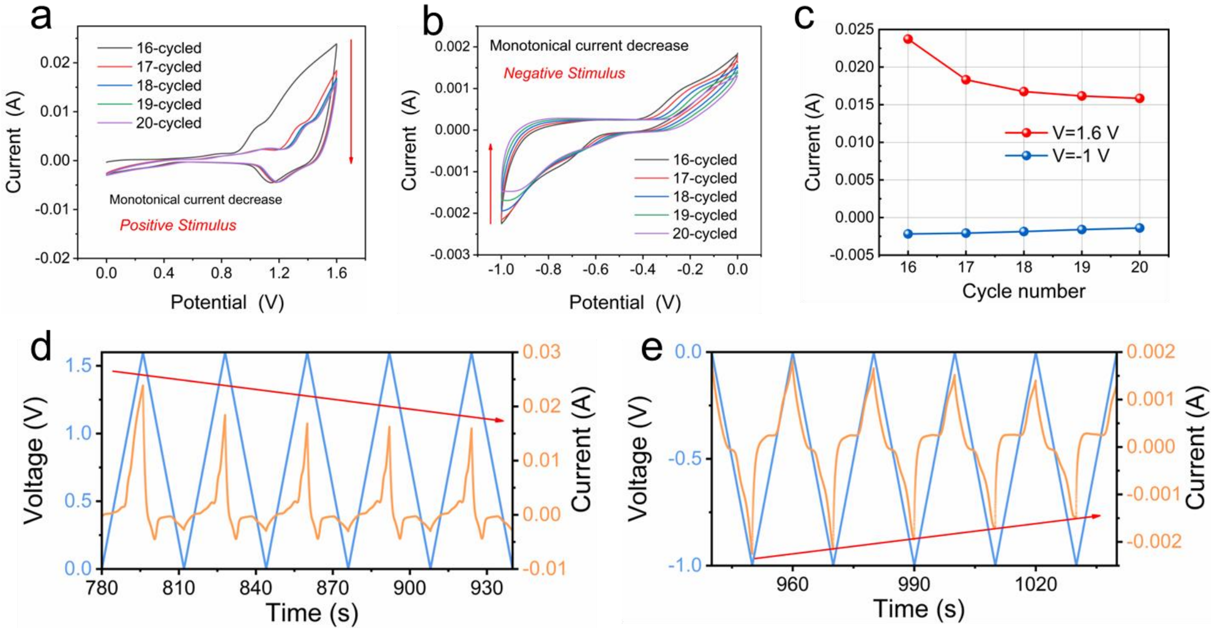

Fig. S38. I-U characteristics of the CAPistor from 16th to 20th cycles. (a, b) The independent I-V characteristics of CAPistor at positive voltage bias (0→1.6→0 V) and negative voltage bias (0→−1.0→0 V), respectively. (c) Current variations at 1.6 or −1.0 V obtained from (a) and (b) are plotted as a function of cycle numbers. (d, e) Evolution of the current (orange) under positive and negative voltage pulses of constant polarity (blue).

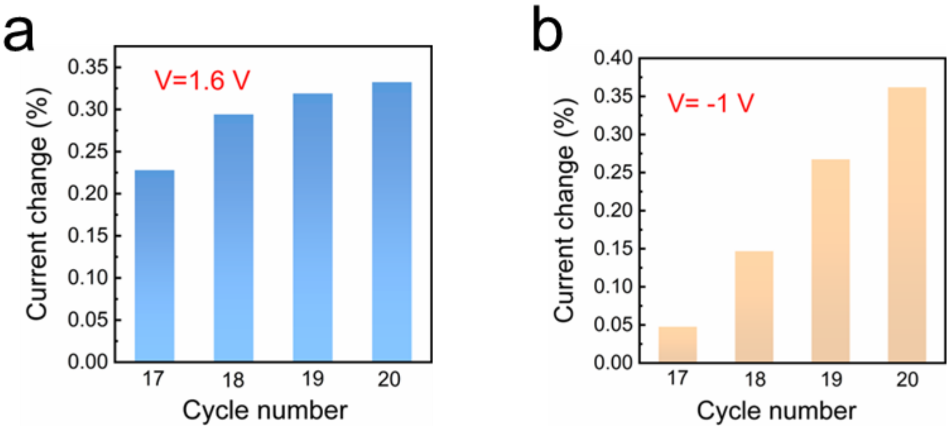

Fig. S39. (a) Current change from 16th to 20th cycles under continuous negative voltage stimulus (V= −1 V). (b) Current change from 16th to 20th cycles under continuous positive voltage stimulus (V= 1.6 V).

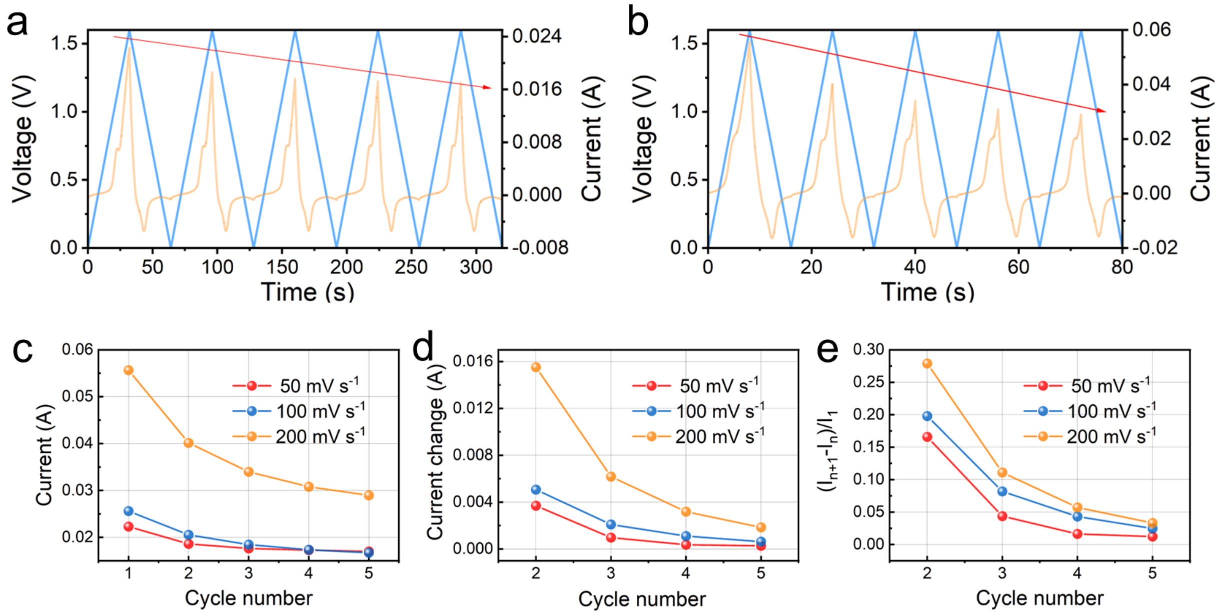

Fig. S40. (a) Evolution of the current (orange) under positive and negative voltage pulses of constant polarity (blue) at 50 mV s^{-1}. (b) Evolution of the current (orange) under positive and negative voltage pulses of constant polarity (blue) at 200 mV s^{-1}. (c) Current values at 1.6 V from 50 mV s^{-1} to 200 mV s^{-1} cycles are plotted as a function of cycle numbers. (d) Current variations at 1.6 V from 50 mV s^{-1} to 200 mV s^{-1} cycles are plotted as a function of cycle numbers. (e) Current decrease ratio from 50 mV s^{-1} to 200 mV s^{-1} cycles are plotted as a function of cycle numbers.

Supplementary Note 7:

To simulate short-term plasticity (STP) electrical pulse patterns, we applied paired voltage pulses and recorded AC current peaks consistent with pulse stimulation. As shown in Fig. S41a and Fig. S41b, Continuous pulses of +1 V induced current decreases (ΔI= 0.0484 A), called a paired-pulse depression (PPD). In the second cycle the current drops by 0.0332 A, which is smaller than the first cycle, which means that the current drop gradually decreases as the pulse progresses. Furthermore, continuous pulses of −1 V applied similarly induced a drop in current (**Fig. S42**), 0.03045 A in the first cycle and 0.00271 A in the second cycle, exhibiting the same paired-pulse depression as the positive pulse. Thus, we provide preliminary evidence of short-term plasticity in CAPistor, which is usually closely linked to critical computational functions for spatio-temporal information processing in biological systems[21].

In the short-time voltage pulse sequence tests performed on the CAPistor, we applied a pulse voltage of ±1 V with a pulse application time of 0.01 seconds. The results are shown in the **Fig. S43**, where a slight drop in current was observed for both short sequential pulses of +1 V and −1 V. It is worth noting that the degree of current drop induced by this short pulse sequence is significantly smaller compared to the paired voltage pulse test (**Fig. S41**). In addition, the degree of current reduction exhibited varying degrees of variability as the pulse interval is increased from 1 to 3 and then 5 seconds (**Fig. S44-45**).

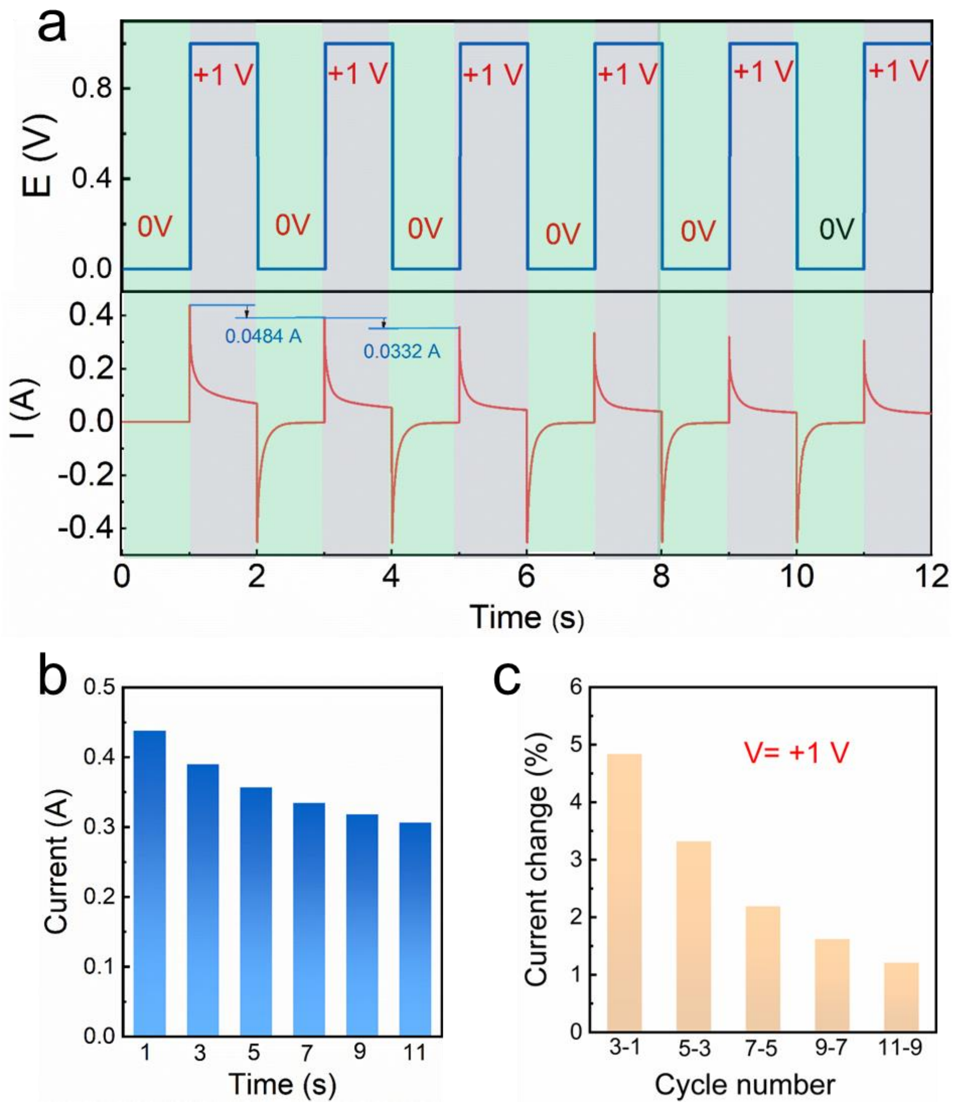

Fig. S41. (a) I-t curve of the CAPistor for an external bias alternating between +1 V and 0 V. (b) The corresponding recorded spike current value. (c) Current difference between neighbouring spikes.

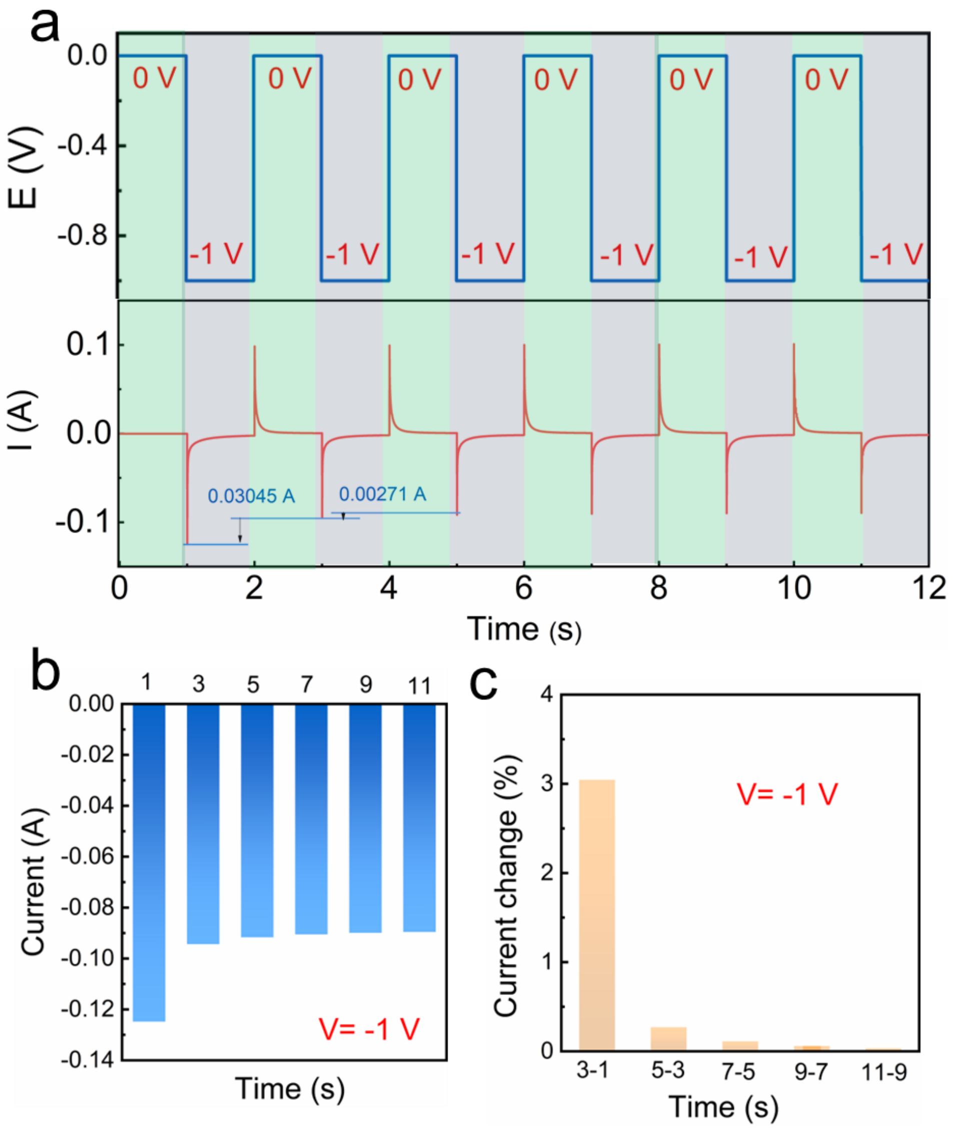

Fig. S42. (a) I-t curve of the CAPistor for an external bias alternating between −1 V and 0 V. (b) The corresponding recorded spike current value. (c) Current difference between neighbouring spikes.

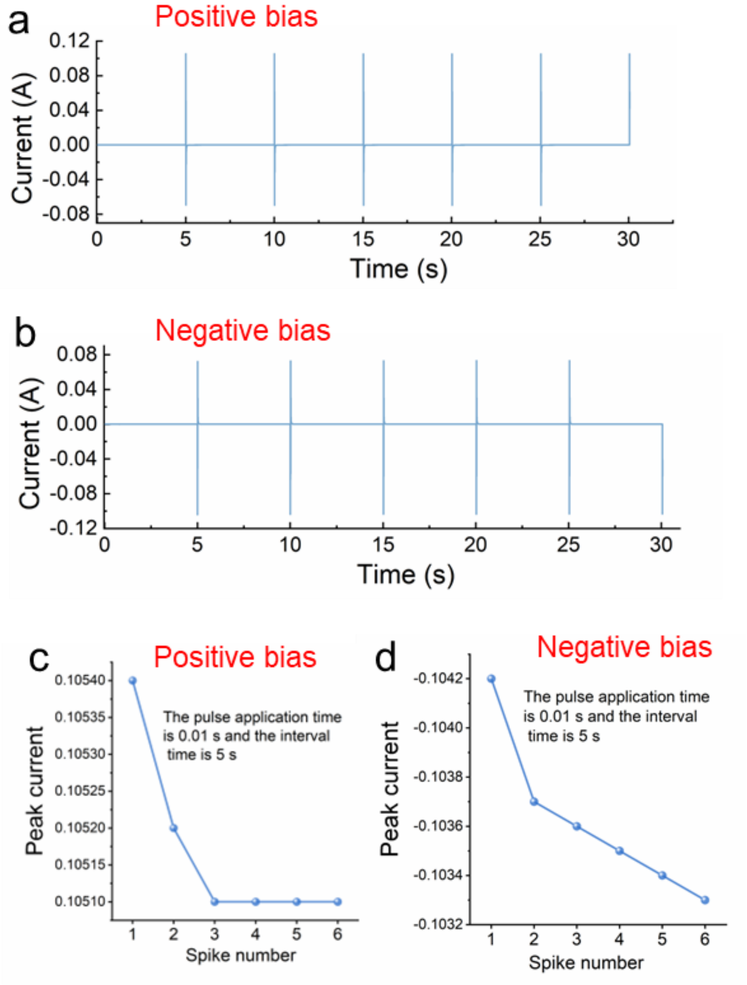

Fig. S43. (a, b) Current responses under voltage pulse train (the pulse voltage is ± 1.0 V and the pulse interval time is 1 s) of the CAPistor with pulse application time of 0.01 s. (c, d) Corresponding variation of the peak current for the corresponding continuous pulse.

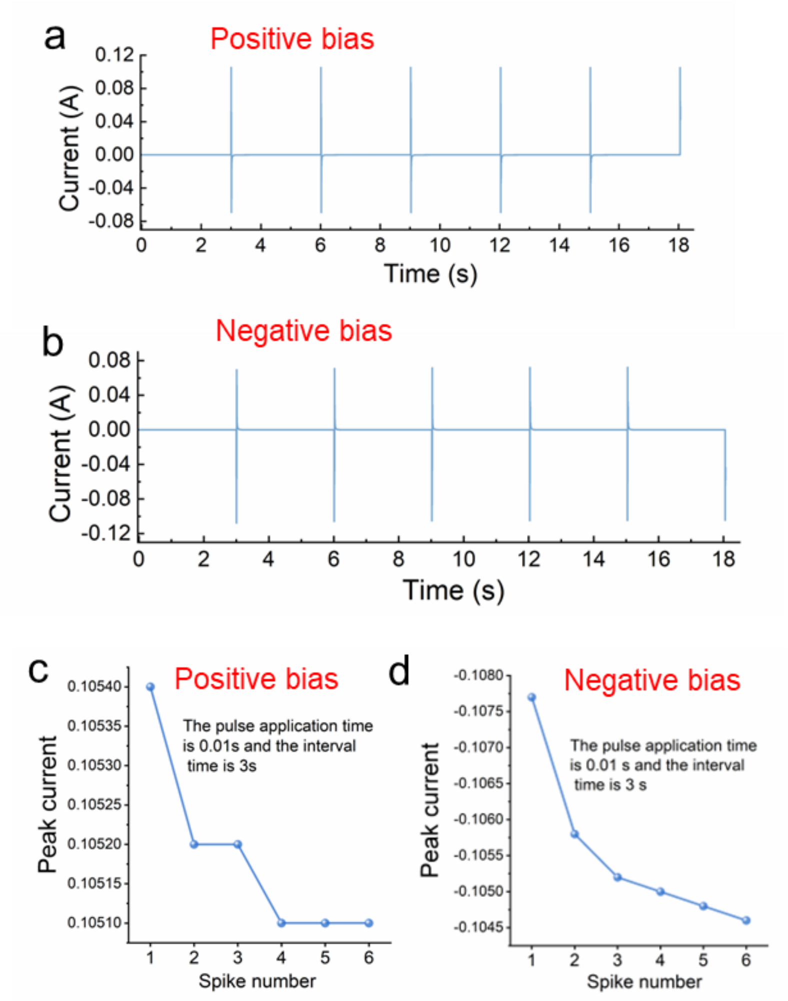

Fig. S44. (a, b) Current responses under voltage pulse train (the pulse voltage is ± 1.0 V and the pulse interval time is 3 s) of the CAPistor with pulse application time of 0.01 s. (c, d) Corresponding variation of the peak current for the corresponding continuous pulse.

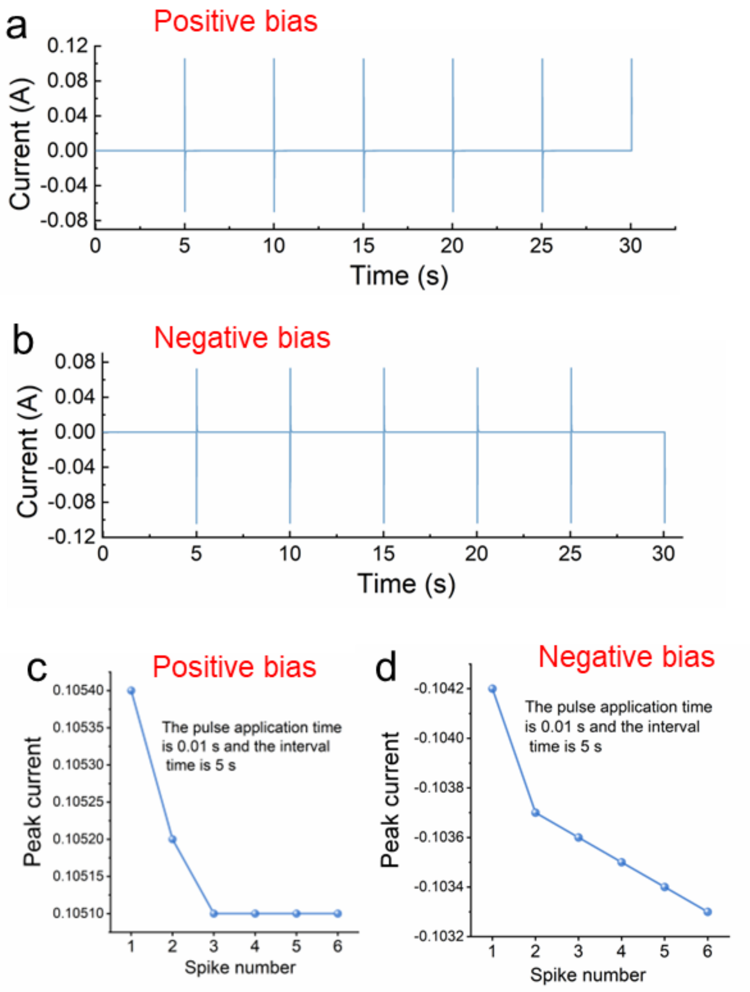

Fig. S45. (a, b) Current responses under voltage pulse train (the pulse voltage is ± 1.0 V and the pulse interval time is 5 s) of the CAPistor with pulse application time of 0.01 s. (c, d) Corresponding variation of the peak current for the corresponding continuous pulse.

Supplementary Note 8:

The key difference between polyelectrolyte-constrained fluidic memristors and supercapacitor memristors lies in the mechanism of charge transport and storage. In the polyelectrolyte-constrained fluidic memristors, the presence of polyelectrolytes introduces ionic interactions and mobility that are sensitive to the direction and magnitude of the applied electric field[21]. This results in a memory effect, where the device "remembers" the previous states of current and voltage, leading to the observed asymmetry in current response under forward and negative biases. However, when a constant voltage is applied to our constructed supercapacitor-memristor, the charge on the surface of the ZIF-7 electrode gradually accumulates, and ions in the electrolyte are arranged near the electrode to undergo a redox reaction, resulting in charging. Initially, the current is high due to the rapid movement of ions to the electrode surface. Over time, the electrode surface charge becomes saturated and the current begins to decrease (**Fig. S46**).

To summarize, the rise in current with forward bias and fall with negative bias in polyelectrolyte-constrained fluidic memristors are manifestations of the memory effect, driven by the unique ionic dynamics within the polyelectrolyte matrix. In contrast, supercapacitor memristors exhibit a consistent current decay regardless of bias direction, due to their reliance on electrostatic charge storage.

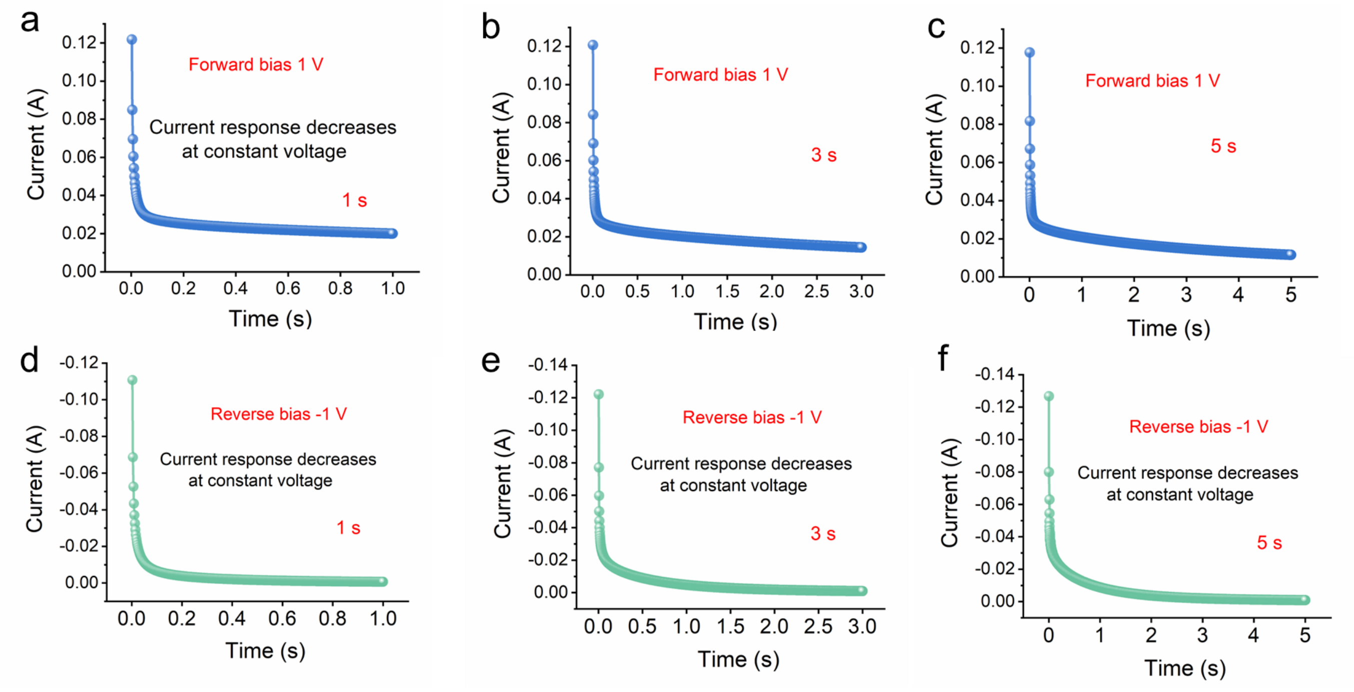

Fig. S46. Current response of CAPistor at different bias voltages. (a-c) Current response at a forward bias voltage of +1 V. (d-f) Current response at a reverse bias voltage of -1 V.

Table S1. Summary of the switching performances of some reported solid state memristor.

Device structure	Mechanism	On/Off ratio	Cyclic number	Retention [s]	References
Au/ferritin/Au	Valence state alternation	<10	<10	10	[22]
Ag/sericin/Au	Charge trap/detrap	10^6	21	10^3	[23]
Ag/leaves/Ti/PET	Metalic filament	50	100	10^3	[24]
Al/gel[24]atin/ITO	Carbon filament	10^6	120	10^5	[25]
Ag/fibroin/Au	Ag filament	＞10^7	20	10^4	[26]
Mg/egg albumen/W	Mg filament	10^3	120	10^4	[27]
Mg/fibroin/Mg	Mg filament	10^3	50	10^4	[28]
Cu/rDnaJ/Pt	Cu filament	10^6	100	10^6	[29]
Al/CDs-silk protein/ITO	Charge trap/detrap	10^6	100	10^6	[30]
Ag/AgNCs@BSA/ITO	Ag filament	10^3	100	10^3	[31]
Mg/Ag-doped chitosan/Mg	Metallic filament	10^2	60	10^4	[32]
Ag/Ag-doped chitosan/Pt	Ag filament	10^5	100	10^4	[33]
Ag/pectin/FTO	Ag filament	450	100	500	[34]
Ag/Ag-doped CM:κ-car/Pt	Ag filament	10^3	50	10^4	[33]
Al/glucose/Si	Interfacial modulation	10^3	100	10^4	[35]
Ag/banana peel/Ti	Metalic filament	20–60	160	4×10^4	[36]
Ag/melanin/ITO	Ag filament	10^4	50	10^4	[37]
This work	Nonlinear ion transport	5349	100	2.5×10^4	

Table S2. EXAFS fitting parameters at the Zn K–edge for various samples.

Sample	Shell	CN^a	R(Å)^b	σ^2(Å^2)^c	ΔE_0(eV)^d	R factor
Zn foil	Zn-Zn	6*	2.64±0.01	0.0107	-0.1	0.0027
	Zn-Zn	6*	2.82±0.01	0.0325	7.7	0.0103
ZnO	Zn-O	4.0±0.3	1.97±0.01	0.0038	7.1	0.0176
	Zn-Zn	12.0±0.7	3.23±0.01	0.0088	5.8	0.0103
Zif-7 0 V	Zn-N	3.1±0.4	1.97±0.01	0.0030	8.0	0.0171
Zif-7 0.5 V	Zn-N	3.4±0.4	1.95±0.01	0.0056	5.1	0.0150
Zif-7 −0.5 V	Zn-N	3.1±0.3	1.96±0.01	0.0050	6.3	0.0112
Zif-7 −0.5 V	Zn-N	2.0±0.3	1.97±0.01	0.0026	6.7	0.0079

^{a}*CN*, coordination number; ^{b}*R*, distance between absorber and backscatter atoms; $^c\sigma^2$, Debye-Waller factor to account for both thermal and structural disorders; $^d\Delta E_0$, inner potential correction; *R* factor indicates the goodness of the fit. S_0^2 was fixed to 0.805, according to the experimental EXAFS fit of Zn foil by fixing CN as the known crystallographic value. A reasonable range of EXAFS fitting parameters: $0.600 < S_0^2 < 1.000$; $CN > 0$; $\sigma^2 > 0$ Å^2; $|\Delta E_0| < 15$ eV; R factor < 0.02.

Notes and references

1. Wu X, Niknam Shahrak M, Yuan B *et al.* Synthesis and characterization of zeolitic imidazolate framework ZIF-7 for CO_2 and CH_4 separation. *Micropor Mesopor Mat* 2014; **190**: 189-196.
2. Kresse G, Furthmüller J. Efficient iterative schemes for ab initio total-energy calculations using a plane-wave basis set. *Phys Rev B* 1996; **54**: 11169-11186.
3. Perdew JP, Burke K, Ernzerhof M. Generalized Gradient Approximation Made Simple. *Phys Rev Lett* 1996; **77**: 3865-3868.
4. Hammer B, Hansen LB, Nørskov JK. Improved adsorption energetics within density-functional theory using revised Perdew-Burke-Ernzerhof functionals. *Phys Rev B* 1999; **59**: 7413-7421.
5. Grimme S. Semiempirical GGA-type density functional constructed with a long-range dispersion correction. *J Comput Chem* 2006; **27**: 1787-1799.
6. Ravel B, Newville M. ATHENA, ARTEMIS, HEPHAESTUS: data analysis for X-ray absorption spectroscopy using IFEFFIT. *J Synchrotron Radiat* 2005; **12**: 537-541.
7. Zabinsky SI, Rehr JJ, Ankudinov A *et al.* Multiple-scattering calculations of X-ray-absorption spectra. *Phys Rev B* 1995; **52**: 2995-3009.
8. Yi X, Rao AM, Zhou J *et al.* Trimming the Degrees of Freedom via a K^+ Flux Rectifier for Safe and Long-Life Potassium-Ion Batteries. *Nano-Micro Lett* 2023; **15**: 200.
9. Ma X, Wu X, Caro J *et al.* Polymer Composite Membrane with Penetrating ZIF-7 Sheets Displays High Hydrogen Permselectivity. *Angew Chem Int Ed* 2019; **58**: 16156-16160.
10. Zhang X, Yang T, Zhang Y *et al.* Single Zinc Atom Aggregates: Synergetic Interaction to Boost Fast Polysulfide Conversion in Lithium-Sulfur Batteries. *Adv Mater* 2023; **35**: 2208470.
11. Kamali K, Prasad S, Sahoo MK *et al.* Unusual CO_2 Adsorption in ZIF-7: Insight from Raman Spectroscopy and Computational Studies. *Inorg Chem* 2022; **61**: 11571-11580.
12. Zhao P, Lampronti GI, Lloyd GO *et al.* Phase Transitions in Zeolitic Imidazolate Framework 7: The Importance of Framework Flexibility and Guest-Induced Instability. *Chem Mater* 2014; **26**: 1767-1769.
13. Ryder MR, Civalleri B, Bennett TD *et al.* Identifying the Role of Terahertz Vibrations in Metal-Organic Frameworks: From Gate-Opening Phenomenon to Shear-Driven Structural Destabilization. *Phys Rev Lett* 2014; **113**: 215502.
14. Zulkifli MYB, Yao Y, Chen R *et al.* Phase control of ZIF-7 nanoparticles via mechanochemical synthesis. *Chem Commun* 2022; **58**: 12297-12300.
15. Davoodi A, Akhbari K, Alirezvani M. Prolonged release of silver and iodine from ZIF-7 carrier with great antibacterial activity. *CrystEngComm* 2023; **25**: 3931-3942.
16. Gao P, Tang P, Mo Y *et al.* Covalency competition induced selective bond breakage and surface reconstruction in manganese cobaltite towards enhanced electrochemical charge storage. *Green Energy Environ* 2022.
17. Gao P, Zeng Y, Tang P *et al.* Understanding the Synergistic Effects and Structural Evolution of $Co(OH)_2$ and Co_3O_4 toward Boosting Electrochemical Charge Storage. *Adv Funct Mater* 2022; **32**(6): 2108644.

18. Zhang Q, Levi MD, Dou Q *et al.* The Charge Storage Mechanisms of 2D Cation-Intercalated Manganese Oxide in Different Electrolytes. *Adv Energy Mater* 2019; **9**: 1802707.
19. Banda H, Dou J-H, Chen T *et al.* Dual-Ion Intercalation and High Volumetric Capacitance in a Two-Dimensional Non-Porous Coordination Polymer. *Angew Chem Int Ed*. 2021; **60**(52): 27119-27125.
20. Ma H, Chen H, Wu M *et al.* Maximization of Spatial Charge Density: An Approach to Ultrahigh Energy Density of Capacitive Charge Storage. *Angew Chem Int Ed* 2020; **59**(34): 14541-14549.
21. Xiong T, Li C, He X *et al.* Neuromorphic functions with a polyelectrolyte-confined fluidic memristor. *Science* 2023; **379**: 156-161.
22. Meng F, Jiang L, Zheng K *et al.* Protein-Based Memristive Nanodevices. *Small* 2011; **7**: 3016-3020.
23. Wang H, Meng F, Cai Y *et al.* Sericin for Resistance Switching Device with Multilevel Nonvolatile Memory. *Adv Mater* 2013; **25**: 5498-5503.
24. Zheng L, Sun B, Mao S *et al.* Metal Ions Redox Induced Repeatable Nonvolatile Resistive Switching Memory Behavior in Biomaterials. *ACS Appl Bio Mater* 2018; **1**: 496-501.
25. Chang Y-C, Wang Y-H. Resistive Switching Behavior in Gelatin Thin Films for Nonvolatile Memory Application. *ACS Appl Mater Interfaces* 2014; **6**: 5413-5421.
26. Wang H, Du Y, Li Y *et al.* Configurable Resistive Switching between Memory and Threshold Characteristics for Protein-Based Devices. *Adv Funct Mater* 2015; **25**: 3825-3831.
27. He X, Zhang J, Wang W *et al.* Transient Resistive Switching Devices Made from Egg Albumen Dielectrics and Dissolvable Electrodes. *ACS Appl Mater Interfaces* 2016; **8**: 10954-10960.
28. Wang H, Zhu B, Ma X *et al.* Physically Transient Resistive Switching Memory Based on Silk Protein. *Small* 2016; **12**: 2715-2719.
29. Jang SK, Kim S, Salman MS *et al.* Harnessing Recombinant DnaJ Protein as Reversible Metal Chelator for a High-Performance Resistive Switching Device. *Chem Mater*. 2018; **30**(3): 781-788. doi: 10.1021/acs.chemmater.7b04261
30. Lv Z, Wang Y, Chen Z *et al.* Phototunable Biomemory Based on Light-Mediated Charge Trap. *Adv Sci* 2018; **5**: 1800714.
31. Shi C, Wang J, Sushko ML *et al.* Silk Flexible Electronics: From Bombyx mori Silk Ag Nanoclusters Hybrid Materials to Mesoscopic Memristors and Synaptic Emulators. *Adv Funct Mater* 2019; **29**: 1904777.
32. Hosseini NR, Lee J-S. Biocompatible and Flexible Chitosan-Based Resistive Switching Memory with Magnesium Electrodes. *Adv Funct Mater* 2015; **25**: 5586-5592.
33. Kim M-K, Lee J-S. Ultralow Power Consumption Flexible Biomemristors. *ACS Appl Mater Interfaces* 2018; **10**: 10280-10286.
34. Sun B, Zhang X, Zhou G *et al.* An organic nonvolatile resistive switching memory device fabricated with natural pectin from fruit peel. *Org Electron* 2017; **42**: 181-186.
35. Park SP, Tak YJ, Kim HJ *et al.* Analysis of the Bipolar Resistive Switching Behavior of a Biocompatible Glucose Film for Resistive Random Access Memory. *Adv Mater* 2018; **30**: 1800722.

36. Zheng L, Sun B, Mao S *et al.* Metal Ions Redox Induced Repeatable Nonvolatile Resistive Switching Memory Behavior in Biomaterials. *ACS Appl Bio Mater* 2018; **1**: 496-501.
37. Chen M, Lv Z, Qian F *et al.* Phototunable memories and reconfigurable logic applications based on natural melanin. *J Mater Chem C* 2021; **9**: 3569-3577.
